# Quest for Nitrous Oxide-reducing Bacteria Present in an Anammox Biofilm Fed with Nitrous Oxide

**DOI:** 10.1264/jsme2.ME23106

**Published:** 2024-03-28

**Authors:** Kohei Oba, Toshikazu Suenaga, Shohei Yasuda, Megumi Kuroiwa, Tomoyuki Hori, Susanne Lackner, Akihiko Terada

**Affiliations:** 1 Department of Chemical Engineering, Tokyo University of Agriculture and Technology, 2–24–16 Naka-cho, Koganei, Tokyo, 184–8588, Japan; 2 Department of Chemical Engineering, Hiroshima University, 1–4–1 Kagamiyama, Higashi-Hiroshima, Hiroshima, 739–8527, Japan; 3 Global Innovation Research Institute, Tokyo University of Agriculture and Technology, 3–8–1 Harumi-cho, Fuchu, Tokyo, 185–8538, Japan; 4 Civil Engineering, School of Engineering, College of Science and Engineering, University of Galway, Galway H91 TK33, Ireland; 5 Environmental Management Research Institute, National Institute of Advanced Industrial Science and Technology, 16–1 Onogawa, Tsukuba, Ibaraki, 305–8569, Japan; 6 Department of Civil and Environmental Engineering Science, Institute IWAR, Chair of Water and Environmental Biotechnology Technical University of Darmstadt, Franziska-Braun-Straße 7, 64287, Darmstadt, Germany

**Keywords:** nitrous oxide reduction, nitrous oxide reductase gene *nosZ*, anammox, denitrifier

## Abstract

N_2_O-reducing bacteria have been examined and harnessed to develop technologies that reduce the emission of N_2_O, a greenhouse gas produced by biological nitrogen removal. Recent investigations using omics and physiological activity approaches have revealed the ecophysiologies of these bacteria during nitrogen removal. Nevertheless, their involvement in‍ ‍anammox processes remain unclear. Therefore, the present study investigated the identity, genetic potential, and activity‍ ‍of N_2_O reducers in an anammox reactor. We hypothesized that N_2_O is limiting for N_2_O-reducing bacteria‍ ‍and an‍ ‍exogeneous N_2_O supply enriches as-yet-uncultured N_2_O-reducing bacteria. We conducted a 1200-day incubation of N_2_O-reducing bacteria in an anammox consortium using gas-permeable membrane biofilm reactors (MBfRs), which efficiently supply N_2_O in a bubbleless form directly to a biofilm grown on a gas-permeable membrane. A ^15^N tracer test indicated that the supply of N_2_O resulted in an enriched biomass with a higher N_2_O sink potential. Quantitative PCR and 16S rRNA amplicon sequencing revealed Clade II *nosZ* type-carrying N_2_O-reducing bacteria as protagonists of N_2_O sinks. Shotgun metagenomics showed the genetic potentials of the predominant Clade II *nosZ*-carrying bacteria, *Anaerolineae* and *Ignavibacteria* in MBfRs. *Gemmatimonadota* and non-anammox *Planctomycetota* increased their abundance in MBfRs despite their overall lower abundance. The implication of N_2_O as an inhibitory compound scavenging vitamin B12, which is essential for the synthesis of methionine, suggested its limited suppressive effect on the growth of B12-dependent bacteria, including N_2_O reducers. We identified *Dehalococcoidia* and *Clostridia* as predominant N_2_O sinks in an anammox consortium fed exogenous N_2_O because of the higher metabolic potential of vitamin B12-dependent biosynthesis.

Increasing concerns for global environmental protection against nitrogen pollution has led to stricter emission standards and the consideration of biological nitrogen removal processes (McCarty, 2018). Anammox-based systems, *e.g.*, partial nitritation-anammox (PNA), are energy-saving and low-cost nitrogen removal processes that represent an alternative to conventional nitrification/denitrification. Since PNA reduces electricity consumption by up to 60% and lowers operation costs due to fewer requirements for aeration and external organic carbon (Gilbert *et al.*, 2015), more than 100 full-scale plants have hitherto implemented PNA in their operation since 2014 (Lackner *et al.*, 2014). The number of anammox-based systems, including PNA, for municipal and industrial wastewater treatment is increasing (Li *et al.*, 2018; Al-Hazmi *et al.*, 2023).

One of the challenges associated with anammox-based systems is the emission of nitrous oxide (N_2_O). N_2_O has a 273-fold higher global warming potential than CO_2_ and is also known as an ozone-depleting substance (Smith *et al.*, 2021). Partial nitritation, the first step of PNA, is a hotspot for N_2_O exhaustion triggered by low oxygen, but high nitrite concentrations (Domingo-Felez and Smets, 2019). Intensive N_2_O emissions potentially offset the reduction in energy consumption and, in some cases, contrarily exceed the carbon footprint required for conventional nitrification/denitrification (Fenu *et al.*, 2019).

Aeration control is a common strategy to deter N_2_O production in conventional wastewater treatment processes (Domingo-Felez and Smets, 2019; Duan *et al.*, 2021). This precautionary strategy is based on preventing the accumulation of nitrite, the primary source of N_2_O production by ammonia-oxidizing and denitrifying bacteria (Peng *et al.*, 2017). Nevertheless, this control measure is not a panacea, resulting in N_2_O emissions in some cases (Han *et al.*, 2023). In contrast, strategies that reduce N_2_O produced via biotic and abiotic pathways have been proposed to mitigate N_2_O emissions. In one countermeasure strategy, gaseous N_2_O in an off-gas line was fed to a bio-scrubber or biofilter, in which N_2_O was converted into harmless nitrogen gas by N_2_O-reducing bacteria (Frutos *et al.*, 2016; Yoon *et al.*, 2017; Han *et al.*, 2023). This concept has been successfully demonstrated in lab- (Yoon *et al.*, 2017) and pilot-scale studies (Han *et al.*, 2023), and is a promising option to reduce N_2_O emissions from wastewater treatment plants (WWTPs) (Frutos *et al.*, 2016; Yoon *et al.*, 2017). In both strategies, harnessing N_2_O-reducing bacteria is vital to accomplish the reduction of N_2_O.

Regardless of the broad range of nitrogen loads, a consistent line-up of bacteria, *a.k.a.* the core microbiome, has been detected (Lawson *et al.*, 2017; Keren *et al.*, 2020; Xiao *et al.*, 2021). Furthermore, non-denitrifying N_2_O-reducing bacteria, which are promising candidates as N_2_O sinks and do not possess either or both nitrite reductase and nitric oxide reductase (Sanford *et al.*, 2012; Shan *et al.*, 2021), have been identified (Lawson *et al.*, 2017). In the core microbiome, some N_2_O-reducing bacteria, consisting of Clade I and Clade II types, in an anammox biomass exhibited high activities devoid of external organic carbon sources as broadly available electron donors (Suenaga *et al.*, 2021). Based on their transcriptional activities, *Anaerolineaceae* (Clade II) and *Burkholderiaceae* (Clade I), counted as part of the core microbiome (Xiao *et al.*, 2021), potentially play an essential role in N_2_O consumption in anammox reactors (Suenaga *et al.*, 2021). A metagenomic approach provides a more detailed understanding of the ecological and physiological functions of the core microbiome. Previous studies elucidated metabolic potentials (Speth *et al.*, 2016; Lawson *et al.*, 2017; Oshiki *et al.*, 2022a) by tracking the uptake of carbon labeled with radioactive and stable isotopes (^14^C [Kindaichi *et al.*, 2012] and ^13^C [Lawson *et al.*, 2021]), indicating the interdependence between anammox and heterotrophic bacteria in carbon metabolism, *e.g.*, vitamins, in anammox reactors (Lawson *et al.*, 2017; Keren *et al.*, 2020; Xiao *et al.*, 2021). However, this physiological interaction of N_2_O-reducing bacteria with carbon and nitrogen compounds remains unclear and, thus, warrants further study.

Since N_2_O-reducing bacteria use N_2_O as an electron acceptor, an external supply of N_2_O may promote the activity of N_2_O-reducing bacteria when N_2_O is a limiting factor. On the other hand, N_2_O potentially inhibits bacterial growth, *e.g.*, *Paracoccus denitrificans*, because it reacts with vitamin B12 and deters methionine biosynthesis initiated from vitamin B12 at extracellular N_2_O concentrations >2.8‍ ‍mg N L^–1^ (Sullivan *et al.*, 2013). This concentration range was observed in an up-flow column reactor (Suenaga *et al.*, 2021) and the anaerobic regions of anammox granules (Okabe *et al.*, 2011). This suppression may be crucial in an anammox community in which most bacteria are interdependent on amino acids and vitamins provided by anammox bacteria (Keren *et al.*, 2020), particularly vitamin B12 (Lawson *et al.*, 2017; Oshiki *et al.*, 2022a). Under these conditions, the supply and retention of additional exogenous N_2_O is a potential inhibitor. These controversial effects of N_2_O on N_2_O-reducing bacteria in an anammox community warrant thorough investigation with the goal of mitigating N_2_O emissions from anammox-based systems.

Therefore, the present study attempted to identify the phylogeny of N_2_O-reducing bacteria in an anammox reactor and characterize their metabolic functions based on their genotypes. Since the growth of N_2_O-reducing bacteria in an anammox community is limited by the supply of N_2_O (Suenaga *et al.*, 2021), we hypothesize that an external N_2_O supply leads to the dominance of fast-growing N_2_O-reducing bacteria under autotrophic conditions. To verify this hypothesis, we operated bioreactors designed to supply sufficient N_2_O without bubble formation via a gas-permeable membrane (Kinh *et al.*, 2017; Suenaga *et al.*, 2019). We examined the effects of the exogenous N_2_O supply on microbial community compositions and functions by the side-by-side operation of bioreactors with or without a N_2_O supply with synthetic media containing ammonia and nitrite. 16S ribosomal RNA (rRNA) gene amplicon sequencing and shotgun metagenomic sequencing were performed for this evaluation.

## Materials and Methods

### Reactor set-up and operation

Two membrane biofilm reactors (MBfRs) (Suenaga *et al.*, 2019) were developed and applied for the enrichment of N_2_O-reducing bacteria (Fig. S1). Each MBfR had liquid and gas compartments between which a flat-sheet silicon gas-permeable membrane was inserted. Their volume (0.26 L) and dimensions are referred to in a previous study (Kinh *et al.*, 2017). One MBfR, Reactor 1 (w/N_2_O), was supplied 5% (v/v) N_2_O (base gas: N_2_) at 5 kPa as a feeding gas from the gas compartment (on the bottom) to the biomass grown on a flat-sheet gas-permeable silicone membrane (L×W of 170×30‍ ‍mm with a wall thickness of 1‍ ‍mm; Rubber) (Fig. S1). The other MBfR, Reactor 2 (w/o N_2_O), had the same configuration and dimensions, but was not supplied with N_2_O. A biomass from an up-flow column-bed anammox reactor (Suenaga *et al.*, 2021) was inoculated into Reactors 1 and 2.

The MBfRs were operated in a thermostatic chamber at 30°C for 1237 days. The medium was continuously supplied, and the liquid was recirculated through ports on the side walls of the MBfRs (Fig. S1). The medium used contained (L^–1^ of distilled water) 100‍ ‍mg N of NH_4_^+^, 100‍ ‍mg N of NO_2_^–^, 540‍ ‍mg of NaHCO_3_, 27‍ ‍mg of KH_2_PO_4_, 300‍ ‍mg of MgSO_4_·7H_2_O, and 180‍ ‍mg of CaCl_2_·2H_2_O. One milliliter of trace element solutions with compositions described elsewhere (deGraaf *et al.*, 1996) was added to per liter of the medium. The mixed medium was continuously purged with N_2_ gas to eliminate dissolved oxygen, and oxygen in the medium influent tank was maintained at a low concentration. The hydraulic retention time (HRT) was consistently set at 1 day.

A sample was taken from the influent and effluent ports (Fig. S1) and stored after filtration through a 0.45-μm membrane filter (A045A025A; Advantec). NH_4_^+^, NO_2_^–^, and NO_3_^–^ concentrations were measured by ion chromatography (ICS1000 and ICS90; Thermo Fisher Scientific). In addition, 1‍ ‍mL of the mixed medium was sampled into a gas vial (13‍ ‍mL) sealed with a butyl rubber stopper filled with 12‍ ‍mL of pure nitrogen gas to measure the gaseous concentration of N_2_O. The dissolved concentration of N_2_O was assessed by the liquid-gas equilibrium method, as previously reported (Isobe *et al.*, 2011; Riya *et al.*, 2012; Suenaga *et al.*, 2021), collecting a gaseous sample for the measurement. The concentration of N_2_O in the headspace was measured by gas chromatography-quadrupole mass spectrometry (GCMS-QP2010 Ultra; Shimadzu).

### Batch test using a ^15^N-labeled tracer

A ^15^N tracer test was performed using the biomass collected from Reactors 1 and 2 on day 776 to evaluate gross anammox, N_2_O production, and N_2_O consumption activities. In the evaluation, 40‍ ‍mg N L^–1^ of ^15^N-labeled ^15^NO_2_^–^ (98% labeled; Shoko Science) and 40‍ ‍mg N L^–1^ of unlabeled NH_4_^+^ were used. Fifteen milliliters of the mixed medium, the same volume as that supplied to the MBfRs, was poured into a 30-mL vial. Approximately 0.3‍ ‍g of the dewatered biomass was inoculated, and ^44^N_2_O was added to evaluate the N_2_O consumption potential. N_2_O and N_2_ concentrations in the headspace were measured by GCMS. Experiments were conducted in triplicate. The biomass pretreatment and measurement conditions are described in our previous study (Suenaga *et al.*, 2021).

Since ^15^NO_2_^–^ contained 98% of labeled ^15^N (^15^N fraction: *F*=0.98), it was assumed that ^15^NO_2_^–^ was converted to ^30^N_2_ via ^46^N_2_O mediated by a heterotrophic denitrifying pathway and ^15^NO_2_^–^ and non-labeled NH_4_^+^ were converted into ^29^N_2_ by the anammox reaction. N_2_O production, N_2_O consumption, and anammox activities were obtained using Eqs. 2–4. In these equations, *V_obs, 44N2O_*, *V_obs, 46N2O_*, *V_obs_,_ 29N2_*, and *V_obs, 30N2_* (note that *V_obs, 44N2O_* is a negative value) were obtained by the linear approximation of changes in the concentrations of ^44^N_2_O, ^46^N_2_O, ^29^N_2_, and ^30^N_2_ from 4 to 15 h. Detailed calculations are described in our previous study (Suenaga *et al.*, 2021).

$$N_{2}O\;\text{production activity}=\left(V_{obs,30N2}+V_{obs,46N2O}\right)∙F^{-2}$$

(Eq. 1)

$$N_{2}O\;\text{consumption activity}=\;V_{obs,30N2}∙F^{-2}-V_{obs,44N2O}$$

(Eq. 2)

Anammox activity

$$=\;\left(V_{obs,29N2}-2∙V_{obs,30N2}∙F^{-1}\left(1-F\right)\right)\;F^{-1}$$

 (Eq. 3)

### Spatial localization of targeted bacteria by fluorescence *in situ* hybridization (FISH)

Biomass samples on day 1044 were subjected to FISH. The floc biomass was collected from Reactor 1 (w/N_2_O) and Reactor 2 (w/o N_2_O) (Fig. S2), followed by immediate fixation with 4% paraformaldehyde. Fixation and subsequent hybridization procedures were performed as previously described (Terada *et al.*, 2013). The oligonucleotide probes applied and formamide percentages are shown in Table S1. A microscopic ana­lysis was performed using a confocal laser scanning microscope (LSM 900; Carl Zeiss) with a Diode Laser (488, 561, and 640‍ ‍nm) and Airyscan 2. Image processing was performed using ZEN 3.0 (blue edition) (Carl Zeiss) and Imaris 10.0 (Oxford Instruments).

### Taxonomy compositions and functional gene abundance

The biomass was routinely collected for a phylogenetic ana­lysis targeting the 16S rRNA gene and the quantification of functional genes. The targeted genes were the *nirK* and *nirS* genes that encode nitrite reductase, the *cnorB* and *qnorB* genes that encode nitric oxide reductase, and the Clade I *nosZ* and Clade II *nosZ* genes that encode N_2_O reductase (NOS). DNA extraction was conducted using the FastDNA Spin Kit for Soil (MP Biomedicals) according to the manufacturer’s instructions. Functional gene quantification was performed using the CFX96 Real-Time PCR Detection System (BioRad Laboratories). The corresponding primers and sequences are summarized in Table S2, and PCR conditions are described in the Supplementary Information (SI).

PCR amplification of the V4 hypervariable region of the 16S rRNA gene was conducted using the primer set 515f-806r (Table S2). PCR conditions and library preparation procedures are described in SI. The DNA libraries generated and the initial control (bacteriophage PhiX; Illumina) were sequenced with a 300-cycle MiSeq Reagent kit (version 2, Illumina) using a MiSeq DNA sequencer (Illumina) in the paired-end sequencing mode.

Sequence data were analyzed using the following bioinformatics tools: removal of the adapter and low-quality reads were conducted using BBMap/BBduk (v. 38.84) (Bushnell, 2014), and trimmed reads were merged using FLASh (version: 2.2.00) (Magoc and Salzberg, 2011). Merged reads were imported into Dada2 (Prodan *et al.*, 2020) (version: 1.26.0). PhiX sequences were removed from imported reads with Dada2’s FilterAndTrim command, and the reminders were then clustered into amplicon sequence variant (ASV) inference (default parameters), followed by the elimination of chimeras (default parameters). Samples with yields >10000 reads in total after the elimination of chimeras were used for a downstream ana­lysis. 16S rRNA ASVs imported into Qiime2 (Bolyen *et al.*, 2019) (q2cli: 2023.5.1) were assigned by lineages using the QIIME 2 q2-feature-classifier plugin (Bokulich *et al.*, 2018) with the pre-trained Silva (138.1) database (Quast *et al.*, 2013; Robeson *et al.*, 2021). Diversity and statistical ana­lyses were conducted using R version 4.2.2 (2022-10-31) and the phyloseq package (McMurdie and Holmes, 2013). The parameters used in the ana­lysis are listed in SI and Table S3-S6.

### Reconstruction and ana­lyses of the metagenome-assembled genome

Three biomass samples, two from Reactor 1 (w/N_2_O) and one from Reactor 2 (w/o N_2_O) were collected on day 981 for shotgun metagenomic sequencing. DNA was extracted using a phenol-chloroform method (Butler, 2012; Yasuda *et al.*, 2020). Biomass samples were centrifuged (10000‍ ‍rpm), and TE buffer (10‍ ‍mM Tris/HCl and 10‍ ‍mM EDTA, pH=8) and 10% SDS were added to the pelleted biomass. Genomic DNA was purified by repeating DNA and protein separation using phenol, chloroform, and CTAB/NaCl solution. RNA as a contaminant in genomic DNA was decomposed by RNaseA (TaKaRa Bio). After ethanol precipitation, genomic DNA was suspended in TE buffer and stored in a freezer until used. Library preparation and sequencing were performed at Azenta Life Science. Sequencing was performed on a Novaseq (Illumina) with a 150-bp paired-end sequencing protocol, and 10 Gb of data was obtained per sample.

The metagenomic pipeline employed in the present study is shown in Fig. S3. A quality check of raw sequencing data and low-quality read trimming were performed using fastp v0.22.0 (Chen *et al.*, 2018). Trimmed reads were assembled by Megahit (v1.2.9) (Li *et al.*, 2015) and metaSPAdes (v3.13.1) (Nurk *et al.*, 2017) in parallel with default parameters. Contigs and scaffolds were filtered using SeqKit (v2.2.0) (Shen *et al.*, 2016), and those longer than 500 bp were used in subsequent ana­lyses.

To analyze the genomic profile of the *nosZ* gene in metagenome samples, gene predictions in filtered contigs and scaffolds were performed by Prodigal (v. 2.6.3) (Hyatt *et al.*, 2010). Predicted genes from all biomass samples were integrated to be non-redundant by Cd-hit (v. 4.8.1) (Li and Godzik, 2006) with ‘-c 0.95‍ ‍-aS 0.9 -g 1’. Function assignments were conducted using EggNOG mapper (v2.1.9) (Cantalapiedra *et al.*, 2021) with the diamond mode and InterProScan (v. 5.65–97.0) (Jones *et al.*, 2014; Blum *et al.*, 2021). Quality-trimmed reads were mapped onto the assembled contigs using BWA-MEM (v. 2.2.1) (Vasimuddin *et al.*, 2019) with default parameters. The resulting sequence alignment mapping files were sorted using SAMtools (v. 1.13) (Danecek *et al.*, 2021), and the read coverage of each contig was calculated using featureCounts (v. 2.0.3) (Liao *et al.*, 2013). Read coverage was further normalized by the total number of mapped reads in each sample, yielding reads per kilobase per million mapped reads (RPKM) values.

To reconstruct the metagenome-assembled genome (MAG), filtered contigs and scaffolds were grouped into primary-bins with MaxBin (v2.2.6) (Wu *et al.*, 2016), MetaBAT (v2.12.1) (Kang *et al.*, 2019), and CONCOCT (v 1.0.0) (Wu *et al.*, 2016) with default parameters. The bins were consolidated into final bins by DASTools (v1.1.4) (Sieber *et al.*, 2018) using default parameters.

Redundant bins generated in parallel were de-replicated by dRep (v3.3.1) (Olm *et al.*, 2017) with ‘-l 50000 -pa 0.90 -sa 0.99 -comp 50 -con 25 -nc 0.1’. CheckM2 (v1.0.1) (Chklovski *et al.*, 2022 CheckM2: a rapid, scalable and accurate tool for assessing microbial genome quality using machine learning. *bioRxiv*: https://doi.org/10.1101/2022.07.11.499243) was used to check the quality of bins, and bins with completeness <70% and contamination >5% were excluded. The presence of 16S/23S/5S rRNA was checked with barrnap (v0.9) (Seemann, 2018), and the numbers and types of tRNAs were counted using tRNAscan-SE (v 2.0.9) (Chan *et al.*, 2021). Taxonomy assignment was conducted using GTDB-Tk (v2.1.1) (Chaumeil *et al.*, 2022) with GTDB r207 (Parks *et al.*, 2022). DFAST (v1.2.14) (Tanizawa *et al.*, 2018) was used for gene predictions and functional annotations. Additional annotations for function assignments were conducted using EggNOG mapper (v2.1.9) (Cantalapiedra *et al.*, 2021) with a diamond mode and KofamScan (v 1.3.0) (Aramaki *et al.*, 2020). Annotation results were merged based on the Kyoto Encyclopedia of Genes and Genomes (KEGG) Orthology (KO) and mapped to the KEGG database (Kanehisa and Goto, 2000) using KEGG Decoder (v1.3) (Graham *et al.*, 2018). The relative abundance of bins in each sample was calculated using CoverM (v0.6.1). Since two biomass samples were collected from Reactor 1 (w/N_2_O), the average relative abundance of the two samples was used.

### Data availability

Raw 16S rRNA gene amplicon and metagenomic sequencing data are available in the DNA Data Bank of Japan (DDBJ) nucleotide sequence database under accession numbers DRA016674 and DRA016675, respectively. Assembled and annotated MAGs were deposited in the DDBJ nucleotide sequence database with the accession numbers shown in Table S7.

## Results

### Reactor operation and nitrogen removal performance

Similar changes were observed in effluent NH_4_^+^, NO_2_^–^, and NO_3_^–^ concentrations in Reactors 1 (w/N_2_O) and 2 (w/o N_2_O) (Fig. S4 and S5). In the first 90 days of the incubation, approximately 50‍ ‍mg N L^–1^ of NH_4_^+^ and NO_2_^–^ remained in the effluent of both reactors. NH_4_^+^ and NO_2_^–^ concentrations in the effluent then decreased in both reactors, reaching average NO_2_^–^ and NH_4_^+^ concentrations of 18.2 and 20.7‍ ‍mg‍ ‍N‍ ‍L^–1^, respectively, from day 100 to 260 in Reactor 1 and 9.76 and 25.1‍ ‍mg N L^–1^, respectively, in Reactor 2. After day 295, NH_4_^+^ and NO_2_^–^ concentrations in the effluent further decreased in Reactors 1 and 2. However, NH_4_^+^ and NO_2_^–^ accumulated in the effluent from day 310 to 405 (Fig. S4b and S5b), possibly due to the lack of maintenance during the lockdown period of COVID-19. After day 426, NO_2_^–^ and NH_4_^+^ concentrations again decreased (16.5 and 12.6‍ ‍mg N L^–1^, respectively, in Reactor 1 and 18.3 and 13.8‍ ‍mg N L^–1^, respectively, in Reactor 2 on average). Influent and effluent pH were 7.90±0.21 and 8.17±0.41, respectively, in Reactor 1 and 7.92±0.31 and 8.13±0.30, respectively, in Reactor 2. The exogenous supply of N_2_O markedly affected dissolved N_2_O concentrations in the bulk liquid. The dissolved concentration of N_2_O in the bulk liquid was 3.96±2.67‍ ‍mg N L^–1^ on average in Reactor 1 with a maximum of 8.95‍ ‍mg N L^–1^ (day 805) and a minimum of 1.31‍ ‍mg N L^–1^ (day 864) (Fig. S6). The dissolved concentration of N_2_O was markedly lower in Reactor 2 (Fig. S7), with an average of 0.67±0.38‍ ‍mg N L^–1^, maximum of 1.72‍ ‍mg N L^–1^ (day 776), and minimum of 0.34‍ ‍mg N L^–1^ (day 1091). Since the total concentration of nitrogen in the influent was 200‍ ‍mg N L^–1^, the N_2_O conversion ratio over total nitrogen in Reactor 2 was 0.34% under the assumption of marginal N_2_O exhaustion to the gaseous layer due to the absence of the headspace, which was similar to that in an anammox column reactor (Suenaga *et al.*, 2021). The stoichiometric ratios of NO_2_^–^ consumption and NO_3_^–^ production over NH_4_^+^ consumption in both reactors were similar to those of enriched anammox bacteria (Strous *et al.*, 1998) (Fig. S4c and S5c), suggesting that an anammox reaction was responsible for biological nitrogen removal.

Regardless of the presence or absence of a N_2_O supply to the reactors, their biomasses were mostly aggregated, consisting of granules or flocs. Although both reactors were constantly stirred by liquid recirculation, some of the biomass sedimented onto the gas-permeable membrane, while the remainder continued to be suspended in the bulk liquid or formed a biofilm on the reactor sidewall or gas-permeable membrane surface (Fig. S2a). However, the biofilm mass of these parts was insufficient to extract DNA (Fig. S2b) for routine functional gene quantification or a phylogenetic ana­lysis. Therefore, samples for DNA extraction were collected from the aggregated biomass in a suspension.

### Intrinsic activity test

**Fig. 1.**
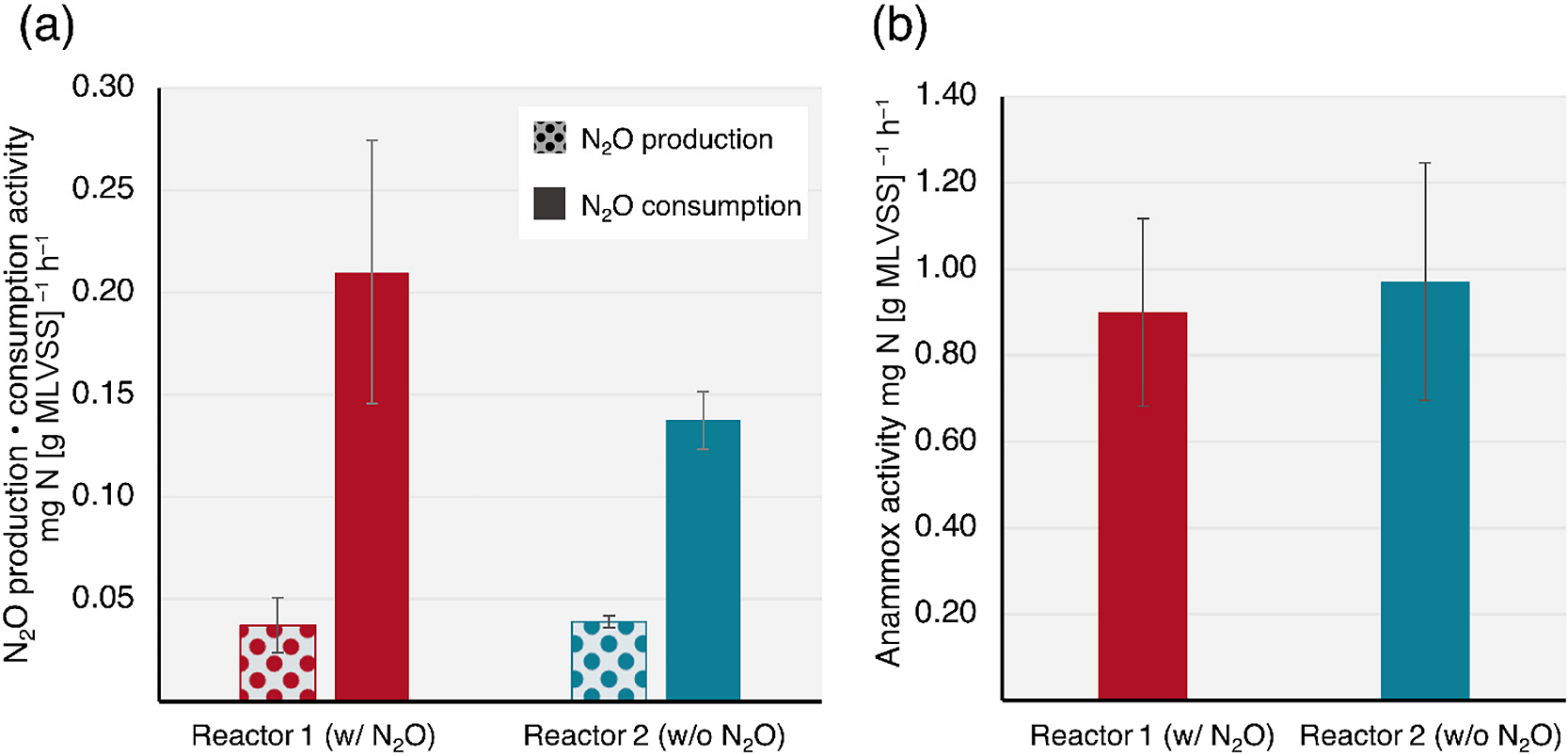
Biomass-specific (a) N_2_O production and N_2_O consumption, (b) anammox activities calculated from the ^15^N tracer test. Experiments were conducted in triplicate for reproducibility. Bars and error bars represent mean values and standard deviations, respectively. In the statistical ana­lysis, Welch’s *t*-test was performed (*P*>0.05).

Biomass-specific anammox rates were 0.90±0.22‍ ‍mg N [g‍ ‍MLVSS]^–1^ h^–1^ for Reactor 1 (w/N_2_O) and 0.97±0.28‍ ‍mg N [g MLVSS]^–1^‍ ‍h^–1^ for Reactor 2 (w/o N_2_O), with no significant difference (*P*=0.735) (Fig. 1). Similar N_2_O production rates were attained: 0.037±0.014‍ ‍mg N [g MLVSS]^–1^ h^–1^ for Reactor 1 (w/N_2_O) and 0.039±0.0028‍ ‍mg N [g MLVSS]^–1^‍ ‍h^–1^ for Reactor 2 (w/o N_2_O). N_2_O consumption rates were 0.21±0.064‍ ‍mg N [g MLVSS]^–1^ h^–1^ for Reactor 1 (w/o N_2_O) and 0.14±0.014‍ ‍mg N [g MLVSS]^–1^ h^–1^ for Reactor 2 (w/o N_2_O). Although no significant differences were observed in N_2_O production (*P*=0.930) or consumption (*P*=0.452), an increase was noted in the N_2_O consumption activity of the biomass fed N_2_O.

### Quantification of functional gene dynamics

**Fig. 2.**
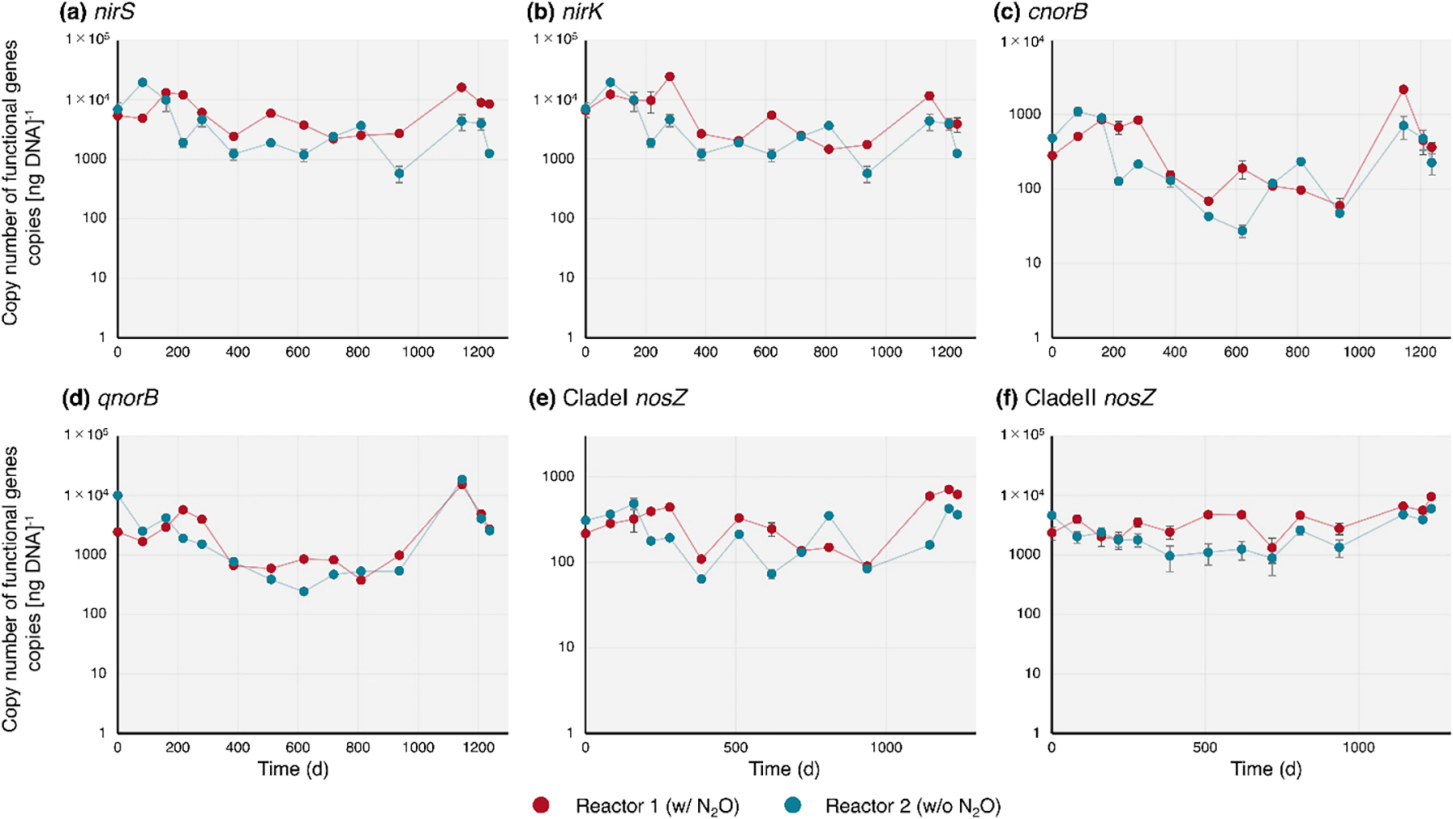
Time courses of gene densities by quantitative PCR (*n*=3): (a) *nirS*, (b) *nirK*, (c) *cnorB*, (d) *qnorB*, (e) Clade I *nosZ*, and (f) Clade II *nosZ* genes normalized by DNA weight.

The dynamics of functional gene abundance are shown in Fig. 2. The continuous external supply of N_2_O allowed for the distinct dynamics of denitrifying genes. Gene copies of *nirK*, *nirS*, *cnorB*, and *qnorB* increased to day 280. Gene copies of *nirK* and *nirS* remained constant in Reactor 1 (w/o N_2_O), while those in Reactor 2 (w/o N_2_O) decreased from those in the inoculum. *nirK* gene abundance was similar in both reactors, whereas that of the *nirS* counterpart was significantly higher in Reactor 1 than in Reactor 2 after day 280, except on days 718 and 810 (*P*<0.05). The continuous external supply of N_2_O increased *nosZ* gene abundance. Despite the presence or absence of the external N_2_O supply, the Clade II *nosZ* gene was one order of magnitude higher than the Clade I *nosZ* gene. In contrast, Clade II *nosZ* gene copies in Reactor 2 (w/o N_2_O) were lower on most sampling dates than those on day 0, which was opposite to that attained in Reactor 1 (w/N_2_O). Clade II *nosZ* gene copies were significantly higher in Reactor 1 than in Reactor 2 after day 280 (*P*<0.05).

### Taxonomy compositions

**Fig. 3.**
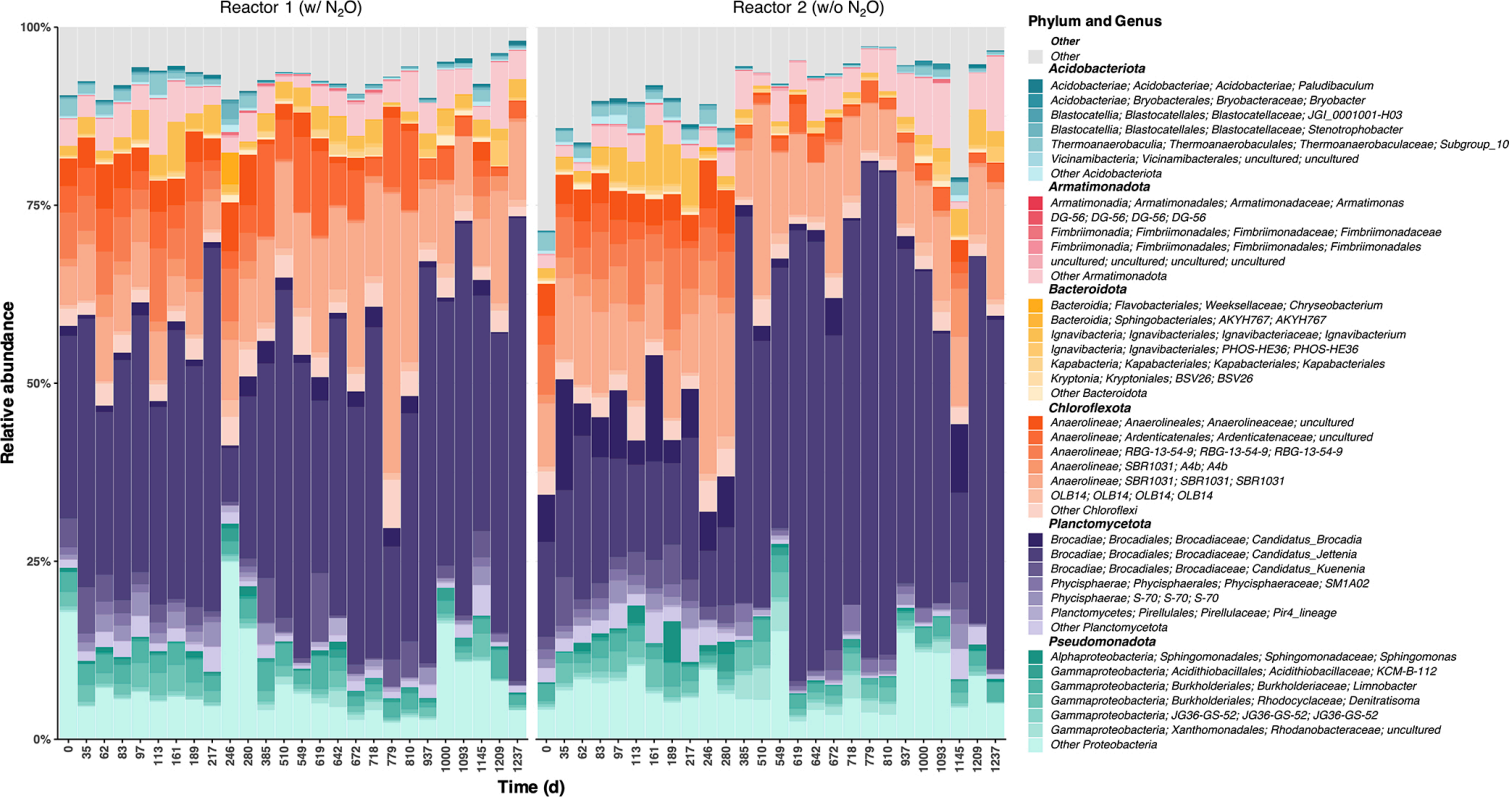
Evolution of microbial community compositions by 16S rRNA gene amplicons in Reactors 1 (w/N_2_O supply) and 2 (w/o N_2_O supply). The top six phyla and genera during the overall incubation are listed.

The top 6 taxa at the phylum level during the entire incubation period are shown in Fig. 3. In both reactors, the phyla *Planctomycetota* and *Chloroflexota* accounted for more than 50% at all periods, except on day 246. The relative abundance of anammox bacteria (class *Brocadiales*) was higher in Reactor 2 than in Reactor 1. The relative abundance of anammox bacteria in Reactor 2 (w/o N_2_O) was on average 24.8% for the period before day 385 and 51.2% after. Within the class *Brocadiales*, *Candidatus* Jettenia was the dominant bacterial species, regardless of the supply of N_2_O.

*Chloroflexota* was the dominant phylum among non-anammox bacteria, with *Anaerolineae* (Reactor 1: 6.3–48.1% of the total, Reactor 2: 5.6–45.6%) and *Ardenticatenaceae* (Reactor 1: 1.9–48.1%, Reactor 2: 5.6–45.6%) as the predominant classes. Other members were‍ ‍*Ignavibacteria*, *Betaproteobacteria*/*Burkholderiales*, *Fimbriimonadia*, *Gammaproteobacteria*/*Xanthomonadales*, and *Gammaproteobacteria*/*Phycisphaerae*. The relative abundance of these non-anammox bacteria, potentially regarded as denitrifiers, was higher in Reactor 1 than in Reactor 2. The relative abundance of non-anammox bacteria changed over time, accounting for an average of approximately 60% and reaching a maximum of 77% in Reactor 1 after day 426, when nitrogen removal performance stabilized.

The bacterial community compositions of the biofilm adhering to the membrane and the aggregate biomass deposited onto the membrane-bound biofilm taken from Reactor 1 on day 199 (Fig. S2) are shown in Fig. S8. The major taxa constituting the community were analogous; however, their compositions differed. *Anaerolineae* was the most dominant biofilm component, of which the ASV assigned to the genus *SBR1031* had a higher relative abundance in the biofilm (32.6%>9.8% in the aggregate biomass). On the other hand, the relative abundance of the family *Brocadiaceae* was lower in the biofilm (3.5%) than in the aggregate biomass (15.7%).

### Activity ana­lysis with FISH imaging

**Fig. 4.**
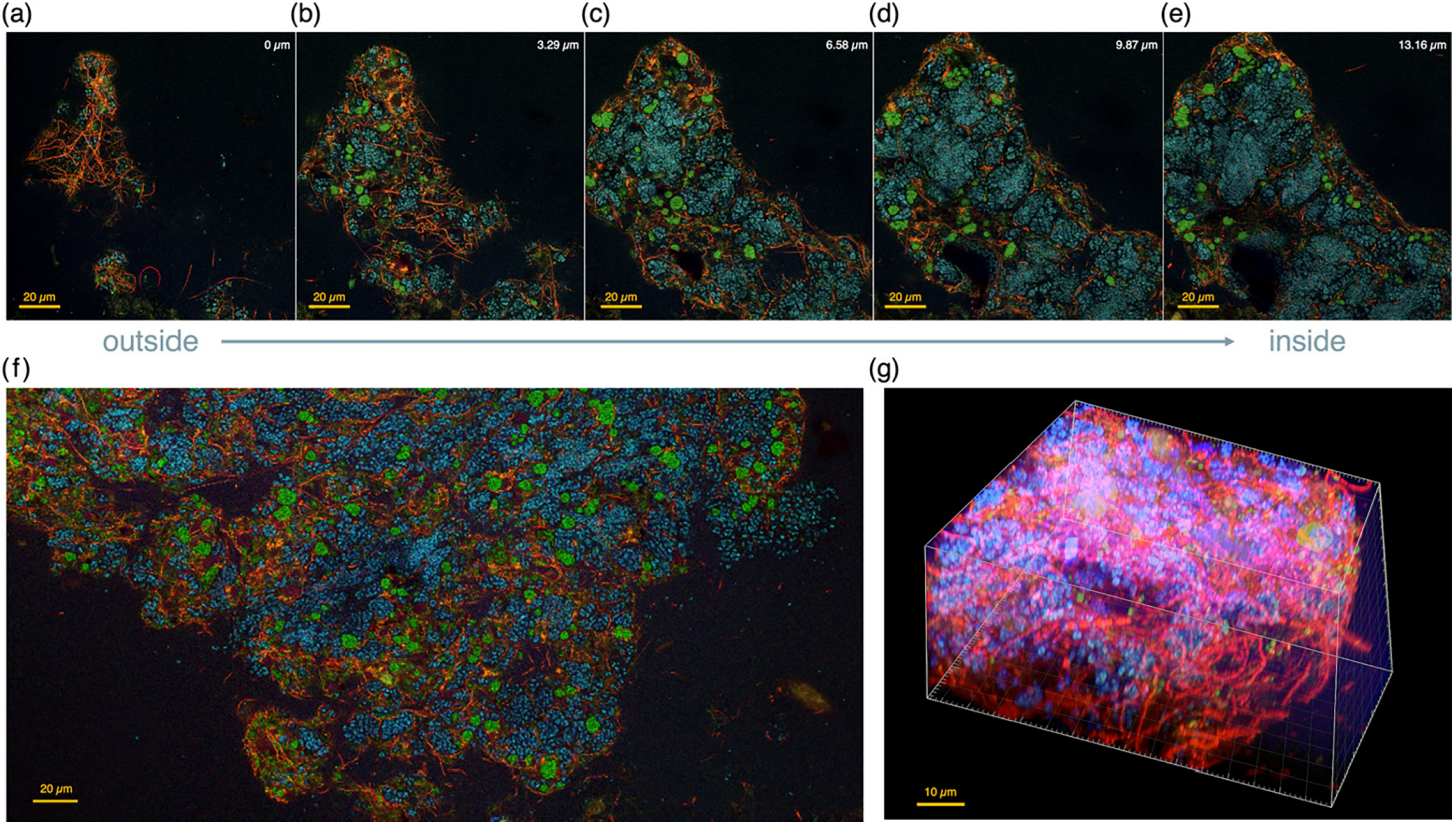
Spatial distributions of anammox and *Chloroflexi* by fluorescence *in situ* hybridization, taken by confocal laser scanning microscopy under 630× magnification. Anammox and *Chloroflexi* were hybridized by probes of Amx 368 and CFX1228+GNSB941 with fluorochromes of Cy5 and Cy3, counterstained by the EUB338 mix with the fluorochrome of FITC. Cy5 is shown in blue, Cy3 in red, and FITC in green. Panels a, b, c, d, e, and f: Reactor 1 (with N_2_O supply). Panel g: Reactor 2 (without N_2_O supply). In Panels a, b, c, d, and e, the number in the upper right in each image indicates the distance from the aggregate surface.

FISH ana­lyses showed sufficient fluorescence in samples taken from Reactor 1 (w/N_2_O) (Fig. 4a, b, c, d, e, f, and g). Consistent with amplicon sequencing results, anammox and *Chloroflexota* were abundant. The spatial distribution of these bacteria in the floc did not markedly differ between the two reactors (data not shown). The mesh-like structure of the phylum *Chloroflexota*, possessing a filamentous cell morphology, was distributed over the floc, particularly in the floc exterior (Fig. 4a, b, c, d, and e). In contrast, anammox bacteria were present inside the floc. Bacteria other than *Chloroflexota* and anammox bacteria were detected near filamentous cells.

### Analyses of MAGs

**Fig. 5.**
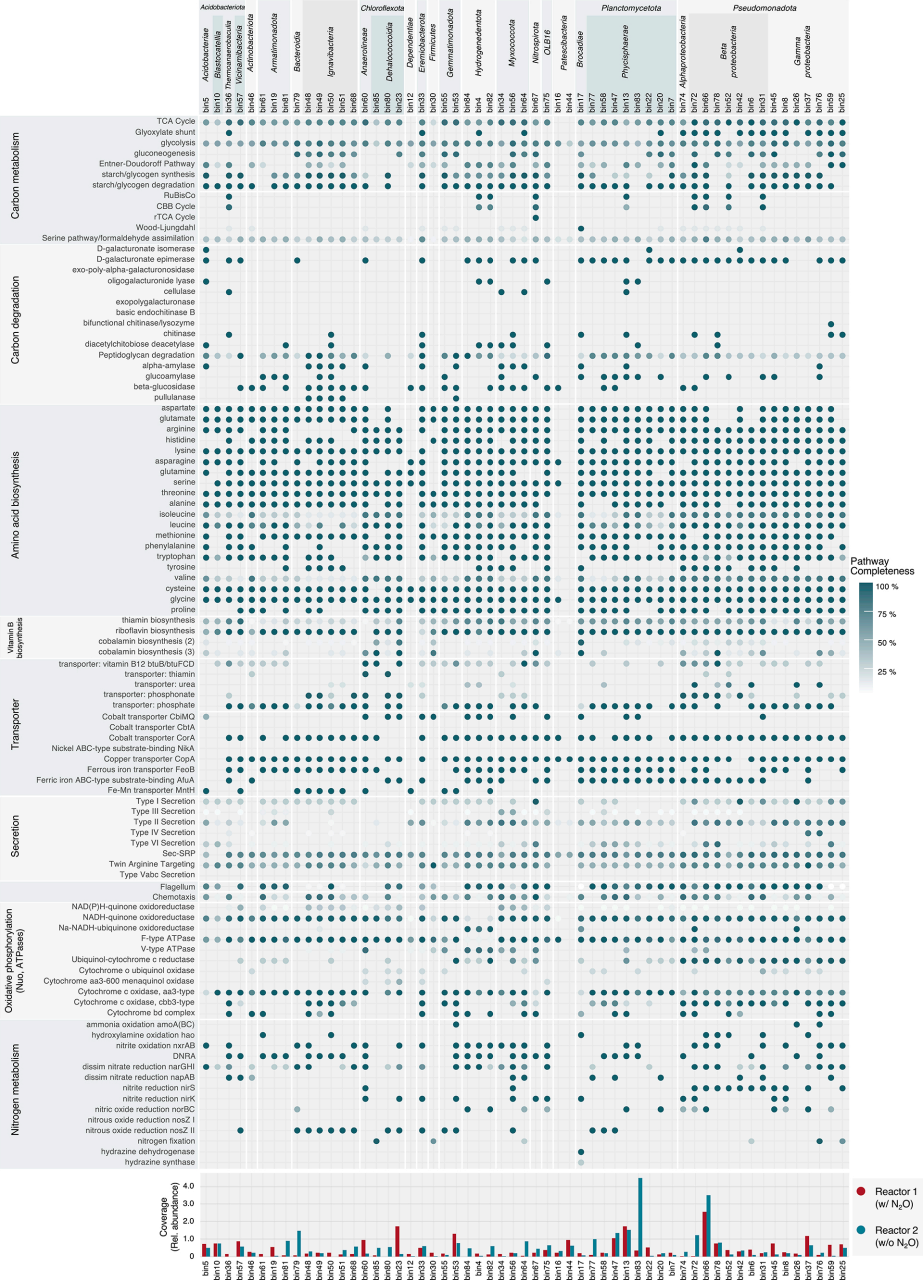
Pathway coverage of high quality (completeness ≥70%, contamination <5%) metagenome-assembled genomes. The dot indicates metabolic pathway completeness calculated using the KEGG Decoder based on KEGG Orthology (KO) results assigned by DFAST, EggNOG mapper, and KofamKOALA.

Of the reconstructed bins, after quality screening (completeness ≥70%, contamination <5%), 58 MAGs were acquired (Fig. 5 and Table S8). MAGs detected with higher‍ ‍relative abundance (>1.5-fold) in Reactor 1 than in Reactor 2 were assigned to *Vicinamibacteria* (bin 57), *Chthonomonadetes* (bin 19), *Anaerolineae* (bin 60), *Dehalococcoidia* (bin 23), *Clostridia* (bin 30), *Gemmatimonadetes* (bins 55 and 53), *Phycisphaerae* (bin 22), *Burkholderiales* (bin 52), *Rhodocyclales* (bin 45), *Nevskiales* (bin 37), and *Xanthomonadales* (bin 59). Abundant MAGs‍ ‍in Reactor 1 did not necessarily harbor either of the‍ ‍*nosZ* genes. It is important to note that all of the retrieved MAGs harboring the *nosZ* gene did not possess a complete set of denitrifying genes, suggesting that they were non-denitrifying genotypes.

MAGs (bins 48, 49, 50, and 51) were affiliated within *Ignavibacteriaceae*, a taxon containing bacteria frequently detected in anammox reactors that has been suggested to function as a N_2_O sink (Lawson *et al.*, 2017) harboring the Clade II *nosZ* gene and dissimilatory nitrate reduction to ammonium (DNRA) gene (*nirBD* and/or *nrfAH*). In addition, bins 48 and 51 carried the *narGHI* genes encoding nitrate reductase. The MAG assigned to *Bacteroidia* (bin 79), which has potential as a N_2_O sink, harbored the Clade II *nosZ* gene and possessed the *norBC* and *narGHI* genes.

*Anaerolineae*, one of the most dominant non-anammox bacterial genera, was annotated with the *narGHI* gene, *nirS* and *nirK* genes, and a group of genes related to DNRA. *Dehalococcoidia* harbored the nitrate reductase *nar* gene,‍ ‍the *nir* gene, and the Clade II *nosZ* gene. *Gemmatimonadetes* (bins 55 and 53), showing a highly transcribed Clade II *nosZ* gene in anammox reactors as previously reported (Suenaga *et al.*, 2021), contained the *nirK* gene encoding nitrite reductase, the *narGHI* gene, and genes‍ ‍involved in DNRA. The MAGs of *Planctomycetota* (bins 77, 58, and 47) were also annotated with the Clade II *nosZ* gene. The MAGs abundantly detected in Reactor 1 (w/N_2_O) did not necessarily harbor any *nosZ* (*i.e.*, bins 19, 60, 6, 45, 37, and 59). The abundance of the *nosZ*-coding sequences collected from the assembled contigs revealed that the higher RPKM values derived from Clade II *nosZ* (174.2 RPKM in Reactor 1 and 164.0 RPKM in Reactor 2) than from Clade I *nosZ* (56.5 RPKM in Reactor 1 and 44.5 RPKM in Reactor 2) were retrieved (Fig. S9).

Genes involved in the metabolism and recycling of peptidoglycans involved in carbon degradation and alpha-amylase and beta-glucosidase, which play a role in the metabolism of polysaccharides, a constitute of extracellular polymeric substances (EPS), were annotated in *Armatimonadota* (bins 61, 19, and 81), *Ignavibacteria*, *Gemmatimonadetes*, *Myxococcota*, and *Planctomycetota*. *Chloroflexota* MAGs differed in their genotypic patterns associated with carbon degradation at the phylum level. For‍ ‍example, the *Anaerolineae* MAG (bin 60) possessed the‍ ‍genes encoding enzymes for peptidoglycan degradation,‍ ‍alpha-amylase and beta-glucosidase, whereas the *Dehalococcoidia* MAG (bin 80) carried an incomplete set of functional genes encoding enzymes for peptidoglycan degradation (Fig. 5).

The genes involved in cobalamin biosynthesis (Corrin ring biosynthesis and adenylation and nucleotide loop assembly) were well annotated in *Dehalococcoidia*, *Clostridia* (bin 30), and *Brocadiae* MAGs, suggesting the presence of important vitamin B12 producers in the anammox community. The coverage of these MAGs was *Dehalococcoidia* (bin 85: 0.08%, bin 23: 1.73%) and *Clostridia* (bin 30: 0.23%). *Burkholderiales* (bins 66, 72, and 78), *Anaerolineae*, and *Nitrospiraceae* (bin 67) MAGs harbored the genes responsible for a vitamin B12 transporter and nucleotide loop assembly for Cobalamin biosynthesis (3) with high annotation (>60%).

## Discussion

Our reactor design with a flat-sheet gas-permeable membrane allowed a bubbleless N_2_O supply to the biomasses in the MBfRs. Continuous incubations by the MBfRs successfully provided environments with high (Reactor 1) and low (Reactor 2) concentrations of N_2_O (Fig. S6 and S7). The concentration of N_2_O in Reactor 1 (3.96‍ ‍mg N L^–1^ on average, *i.e.*, 141‍ ‍μM) was more than one order of magnitude higher than the apparent half-saturation constant for N_2_O (*K*_m_,_N2O_) of 9.50±3.0‍ ‍μM (0.266±0.083‍ ‍mg N L^–1^) of an enriched anammox biomass (Suenaga *et al.*, 2021). Therefore, this ensures an environment in which fast-growing N_2_O reducers with a low affinity for N_2_O, but a high N_2_O consumption rate (Andrews and Harris, 1986; Yin *et al.*, 2022) preferentially grow. This study demonstrated that the application of an MBfR concept (Nerenberg, 2016) to enrich highly efficient N_2_O-reducing bacteria in an anammox biomass secures a stable environment with high or low concentrations of N_2_O with the biomass. The maintenance of a different N_2_O level for a long period was essential in the present study, while we previously conducted a short-term (within 1 day) biomass exposure to a high N_2_O concentration (Suenaga *et al.*, 2021).

By using the MBfR concept, the present study investigated whether an external N_2_O supply led to the dominance of fast-growing N_2_O-reducing bacteria. According to the microbial community compositions in the two MBfRs with and without the external N_2_O supply (Fig. 3), slight changes in microbial community compositions were attained. This result refutes our hypothesis that an external N_2_O supply leads to the dominance of fast-growing N_2_O-reducing bacteria. Nevertheless, the biomass from Reactor 1 showed a higher N_2_O consumption rate than that from Reactor 2 (Fig. 1). The quantitative values of Clade I and Clade II *nosZ* genes indicated that the supply of N_2_O as an external electron acceptor was advantageous to the bacterial community harboring *nosZ*. This effect was significantly pronounced for Clade II *nosZ* (Fig. 2f). Although the contribution of the external N_2_O supply to microbial community changes may be limited (Fig. 3), the N_2_O supply was instrumental in the increases observed in Clade II *nosZ* gene abundance (Fig. 2f) and intrinsic N_2_O consumption activities (Fig. 1).

An autotrophic environment with a high nitrogen concentration likely favors Clade II *nosZ* N_2_O-reducing bacteria. This notion was supported by the present results showing that Clade II *nosZ* N_2_O-reducing bacteria were consistently one order of magnitude and three-fold more abundant than the Clade I type in MBfRs regardless of the supply of N_2_O by qPCR (Fig. 2e and 2f) and the metagenomic ana­lysis (Fig. S9), respectively. This result may be attributed to an inoculum being highly enriched under autotrophic conditions (Suenaga *et al.*, 2021). Previous studies on N_2_O-reducing bacteria harboring either Clade I or Clade II *nosZ* genes indicated that Clade II *nosZ* bacteria exhibited higher affinities (Yoon *et al.*, 2016; Suenaga *et al.*, 2019), which was not consistent with our results, where even a high concentration of N_2_O allowed a higher abundance of Clade II *nosZ* bacteria. Clade II *nosZ* bacteria were found to be dominant during an incubation with a N_2_O supply (Suenaga *et al.*, 2019). These findings and the present results indicate that the concentration of N_2_O during the enrichment of N_2_O-reducing bacteria is not the sole factor affecting the predominance of the clade of the *nosZ* gene.

*Anaerolineae* and *Ignavibacteria*, which were dominant among non-anammox bacteria in the present study (Fig. 3), may be critical N_2_O reducers in anammox systems. They reportedly utilize endogenous organic matter when fed an organic-free medium, and are also the most prevalent when fermentable organic matter is supplied at low C/N ratios (Xiao *et al.*, 2021). *Ignavibacteria* MAGs (bins 48, 49, 50, and 51) and *Anaerolineae* MAG (bin 80) were annotated with metabolic pathways regarding soluble microbial products (SMP) by KEGG Decoder (Ni *et al.*, 2012), including EPS composed of polysaccharides and proteins secreted by anammox bacteria (Hou *et al.*, 2015; Ali *et al.*, 2018). The annotation count by KEGG Decoder accounted for over 65% of the total metabolic pathways. In addition, these associated genes were functionally assigned to carbon degradation to utilize cell debris (Fig. 5). Therefore, these taxa were likely to be N_2_O consumers using SMP produced by anammox and others in the anammox reactor.

*SBR1031* belonging to the class *Anaerolineae* showed a higher relative abundance in the biofilm grown on membrane surfaces (Fig. S8) with presumably high N_2_O concentrations. The results of the metagenomic ana­lysis suggest that this genus contains bacteria harboring the Clade II *nosZ* gene (Bovio-Winkler *et al.*, 2023), which may have a promising N_2_O consumption capacity. Therefore, the class *Anaerolineae* has potential as a N_2_O sink in the anammox system that is not susceptible to high N_2_O concentrations. In contrast, a lower abundance of the family *Brocadiaceae* in the biofilm than the deposited aggregates on the biofilm outer surface (Fig. S8) suggested that a high N_2_O concentration condition was unfavorable for anammox growth.

The phylum *Chloroflexota* coexisting with anammox bacteria was detected at the periphery of the biomass, surrounding anammox cell aggregates (Fig. 4). This spatial arrangement did not appear to change with or without the N_2_O supply, which is consistent with previous findings (Wong *et al.*, 2023). In addition, filamentous *Chloroflexota* has been suggested to function as a junction for biomass aggregates and degrade EPS secreted by anammox bacteria, which are responsible for decomposing EPS to SMP available to other heterotrophic bacteria (Wong *et al.*, 2023). Our *Anaerolineae* MAG (bin 60) was also well annotated with pathways (alpha-amylase and beta-glucosidase) involved in the degradation of polysaccharide chains (Oshiki *et al.*, 2022a) (Fig. 5). Our FISH ana­lysis also showed fluorescence derived from neither *Planctomycetota* nor *Chloroflexota* in the vicinity of *Chloroflexota* (Fig. 4), and its spatial coordination was similar to that observed in a previous study (Wong *et al.*, 2023). Therefore, the preference and availability of recalcitrant organic matter were deemed necessary for elucidating the composition of the non-anammox bacterial population. This unexplored population may be one of the reasons why N_2_O-reducing bacteria with faster N_2_O consumption rates were more dominant than N_2_O-producing bacteria.

Although the phyla *Gemmatimonadota* and non-anammox *Planctomycetota* were not predominant in the microbial community, they were frequently detected taxa in Reactor 1 (Fig. 5), suggesting their involvement in another N_2_O sink. These phyla exhibit high transcription activities of the *nosZ* gene in anammox processes (Park *et al.*, 2017; Suenaga *et al.*, 2021). The higher coverage of MAGs belonging to these strains in Reactor 1 suggested that the physiological traits matched the environment in the MBfR with external N_2_O supply, alluding to N_2_O reducers favorable in environments with high N_2_O concentrations. The production of N_2_O during denitrification, often found in anammox processes as a minor microbial reaction among nitrogen transformation, may be attributed to a N_2_O production rate via the reduction of NO_3_^–^ and NO_2_^–^ to N_2_O, overwhelming the N_2_O consumption rate. A *Gemmatimonadetes* MAG (bin 53), harboring *narGHI* and Clade II *nosZ* genes, possessed the metabolic potentials of NO_3_^–^ and N_2_O reduction (Fig. 5), potentially consuming N_2_O depending on N_2_O concentrations according to the biokinetics in previous study (Oshiki *et al.*, 2022b). Moreover, non-anammox *Planctomycetes* (bins 77 and 47) possessed two denitrification-related genes, the *napAB* gene encoding nitrate reductase and the Clade II *nosZ* gene (Fig. 5). When bacteria carry the *nap* and *nos* genes, they preferentially use N_2_O as an electron acceptor more than those with *nar* and *nos* genes (Gao *et al.*, 2021; Oba *et al.*, 2022), suggesting that non-anammox planctomycetes harboring *nosZ* genes are preferential N_2_O sinks.

Theoretically, there has been a concern about the loss of nitrogen removal performance due to reduced microbial activity caused by N_2_O, which has yet to be examined in detail. Excessive N_2_O in Reactor 1 may react with cob(I)alamin (vitamin B12) as previously reported (Drummond and Matthews, 1994), thereby inhibiting the function of the cobalamin-dependent enzyme (Shelton *et al.*, 2019). The N_2_O level in Reactor 1 (3.96‍ ‍mg N L^–1^) exceeded the concentration (>2.8‍ ‍mg N L^–1^) at which N_2_O reacts with MetH methionine synthase, the most widely used cobalamin-dependent enzyme, thereby retarding the cell growth of *P. denitrificans* (Sullivan *et al.*, 2013). To ameliorate the inhibition of N_2_O, *P. denitrificans* and *Dehalococcoides mccartyi* either activate vitamin B12-independent MetE methionine synthase or maintain its activity by an endogenous and exogenous vitamin B12 supply (Sullivan *et al.*, 2013; Yin *et al.*, 2019). *Bacteroidia* and *Anaerolineae* (bins 79 and 60), predominant taxa based on 16S rRNA gene amplicon sequencing (Fig. 3), did not possess a *metE* gene encoding vitamin B12-independent MetE methionine synthase (Fig. 5). Bacteria possessing only vitamin B12-dependent enzymes may be disadvantageous for survival under excessive N_2_O conditions. Nevertheless, their abundance did not decrease in the presence of excessive N_2_O in Reactor 1 (Fig. 3). The results of the FISH ana­lysis also indicated that the dominance of *Chloroflexota* was sustained (Fig. 4). Therefore, the anammox community in Reactor 1 appeared to have a bypassing mechanism by which vitamin B12 was relayed from its producers to acceptors. Further studies are needed to elucidate the underlying mechanisms.

Previous studies reported that *de novo* cobamide biosynthesis consists of approximately 30 steps, which may be broadly divided into the following steps: tetrapyrrole precursor biosynthesis, (aerobic/anaerobic) corrin ring biosynthesis and adenylation, and nucleotide loop assembly (Shelton *et al.*, 2019; Lu *et al.*, 2020; Balabanova *et al.*, 2021). Furthermore, among an anammox community, only *Brocadia* sp. synthesizes cobalamin (Lawson *et al.*, 2017; Keren *et‍ ‍al.*, 2020). In the present study, only anammox *Planctomycetes* (bin 17) had complete *de novo* biosynthetic pathways, which was consistent with previous findings.

The results obtained herein demonstrated that *Dehalococcoidia* (bins 23 and 85) and *Clostridia* (bin 30) carried a nucleotide loop assembly pathway following the cobalamin precursor synthesis pathway. Some bacteria have been shown to partially possess *de novo* cobalamin biosynthesis pathways (Shelton *et al.*, 2019; Lu *et al.*, 2020). As shown in physiological studies (Schipp *et al.*, 2013; Shelton *et al.*, 2019), some *Dehalococcoidia* and *Clostridia* synthesize cobamides from salvaged precursors or change the cobamide structure for their growth. The results of our metagenomic ana­lysis suggest that Clade II *nosZ* N_2_O-reducing bacteria, such as *Dehalococcoidia* and *Clostridia*, retain many genes associated with the cobalamin synthesis pathway. Putatively, they are prone to endogenously synthesize cobalamin and/or exogenously receive cobalamin supplied by *Brocadia* as a survival strategy. The present results imply the strong contribution of anammox bacteria as a *de novo* cobalamin producer to relay cobalamin to coexisting bacteria missing its production functions, thereby restricting bacteria from cobalamin inhibition caused by N_2_O in the anammox community.

## Conclusion

In the present study, we operated MBfRs that were externally supplied with N_2_O for 1200 days for the continuous incubation of bacteria consuming N_2_O. We demonstrated the long-term maintenance of a high abundance of Clade II *nosZ* and an increased capacity for N_2_O consumption in the community enriched by an exogenous N_2_O supply. On the other hand, slight differences were attained in the dominant taxa in the MBfRs with or without an external N_2_O supply, which indicated that supplying N_2_O as an additional electron acceptor did not necessarily result in the dominance of fast-growing N_2_O-reducing bacteria. Therefore, other factors, particularly electron donors, limited the growth rate of heterotrophic N_2_O-reducing bacteria. Despite growth-limiting conditions for N_2_O-reducing bacteria, the metagenomic ana­lysis revealed significant functions to acquire organic matter and vitamins that serve as electron donors. *Anaerolineae* and *Ignavibacteria*, regarded as parts of the core microbiome and dominant in the MBfR supplied with N_2_O, have a broad availability of organic matter and survive at high N_2_O concentrations. *Dehalococcoidia* and *Clostridia* have partial vitamin B12 production pathways, likely carrying a survival strategy in excessive N_2_O environments. Moreover, we found that non-anammox *Planctomycetes* appeared to contribute to N_2_O consumption despite their low abundance in the anammox community. Since the concentration of N_2_O in the MBfR system supplied with N_2_O was markedly higher than that often observed in anammox reactors, there have been concerns that the anammox community may lose its activity due to the inhibition of N_2_O. Nevertheless, the N_2_O-fed reactor operation underpinned the anammox community, exerting a stable N_2_O sink at excessively high N_2_O concentrations. Our in-depth ana­lysis of microbial community compositions and functions provide insights on the as-yet-unknown functions of non-denitrifying Clade II *nosZ* bacteria. These ecophysiological descriptions will facilitate the utilization of N_2_O-reducing bacteria inhabiting engineered systems that emit N_2_O, such as anammox-based processes, and may be expanded to agricultural fields requiring N_2_O mitigation. The may lead to the development of countermeasures against N_2_O emissions, *e.g.*, bioaugmentation by inoculating biomasses enriching N_2_O reducers.

## Citation

Oba, K., Suenaga, T., Yasuda, S., Kuroiwa, M., Hori, T., Lackner, S., and Terada, A. (2024) Quest for Nitrous Oxide-reducing Bacteria Present in an Anammox Biofilm Fed with Nitrous Oxide. *Microbes Environ ***39**: ME23106.

https://doi.org/10.1264/jsme2.ME23106

## Supplementary Material

Supplementary Material
