# Supplementary material for: Quest for Nitrous Oxide-reducing Bacteria Present in an Anammox Biofilm Fed with Nitrous Oxide
Source: Microbes Environ. 2024 Mar 28;39(1):ME23106. doi: 10.1264/jsme2.ME23106 (PMC10982107; doi:10.1264/jsme2.ME23106)
Supplement: Supplementary file 1 — Supplementary Material [file 39_23106_s1.pdf]

SUPPLEMENTARY INFORMATION

Quest for Nitrous Oxide-reducing Bacteria Present in an Anammox Biofilm Fed with Nitrous Oxide

Kohei Oba[1], Toshikazu Suenaga[2, 3], Shohei Yasuda[3, 4], Megumi Kuroiwa[1], Tomoyuki Hori[5], Susanne Lackner[3,6], Akihiko Terada[1,3]*

1. Department of Chemical Engineering, Tokyo University of Agriculture and Technology, 2-24-16 Naka-Cho, Koganei, Tokyo, 184-8588, Japan
2. Department of Chemical Engineering, Hiroshima University, 1-4-1 Kagamiyama, Higashi-hiroshima, Hiroshima, 739-8527, Japan
3. Global Innovation Research Institute, Tokyo University of Agriculture and Technology, 3-8-1 Harumi-Cho, Fuchu, Tokyo, 185-8538, Japan
4. Civil Engineering, School of Engineering, College of Science and Engineering, University of Galway, Galway H91 TK33, Ireland
5. Environmental Management Research Institute, National Institute of Advanced Industrial Science and Technology, 16-1 Onogawa, Tsukuba, Ibaraki, 305-8569, Japan
6. Department of Civil and Environmental Engineering Science, Institute IWAR, Chair of Water and Environmental Biotechnology, Technical University of Darmstadt, Franziska-Braun-Straße 7, 64287, Darmstadt, Germany

List of Supplementary contents (27 pages in total)

Reactor setup (**Fig. S1** and **S2**) P. S3-4

Conditions and probes of FISH (**Table S1**) P. S5

Conditions of quantitative real-time PCR (**Table S2**) P. S6

Reconstruction and analyses of the metagenome-assembled genome (**Fig. S3**) P. S7-9

Functional gene involved in methionine biosynthesis (**Table S3, S4, and S5**) P. S10-12

Genes defined as involved in peptidoglycan degradation (**Table S6**) P. S13

Fig. S4 Concentration of ionic nitrogen compounds in influent and effluent of Reactor 1 with N_2O supply. P. S14

Fig. S5 Concentration of ionic nitrogen compounds in influent and effluent of Reactor 2 without N_2O supply. P. S15

Fig. S6 Dissolved N_2O concentration in Reactor 1 (N_2O was supplied) P. S16

Fig. S7 Dissolved N_2O concentration in Reactor 2, where N_2O was not supplied P. S16

Fig. S8 Microbial community compositions of the aggregate and biofilm Reactor 1 P. S.17

Fig. S9 Normalized read counts of Clade I and Clade II *nosZ* genes in the assembled contigs. P. S.18

Table S7 DDBJ accession numbers of the metagenome-assembled genomes (MAGs) P. S19–20

Table S8 Quality information on the retrieved MAGs P. S21–22

Table S9 Taxonomy assignment using GTDB-Tk with GTDB (r207) database P. S23–24

References P. S25-27

Reactor setup

The volume of the membrane biofilm reactor (MBfR) was 260 mL, and a flat-sheet silicone gas-permeable membrane (170 mm × 30 mm × 1 mm) (Rubber Co., Tempe, AZ, USA) was installed at the bottom of the reactor. N_2O was supplied to the biomass in the liquid phase through the gas-permeable membrane (**Fig. S1**). The culture medium supply, outflow, and liquid circulation were conducted through ports on the side of an MBfR. Therefore, the medium was gently mixed with circulating liquid.

Biofilms were grown onto the sidewall and the surface of the flat-sheet silicone gas-permeable membrane (**Fig. S2**). Together with biofilm formation, aggregates were deposited onto the membrane.

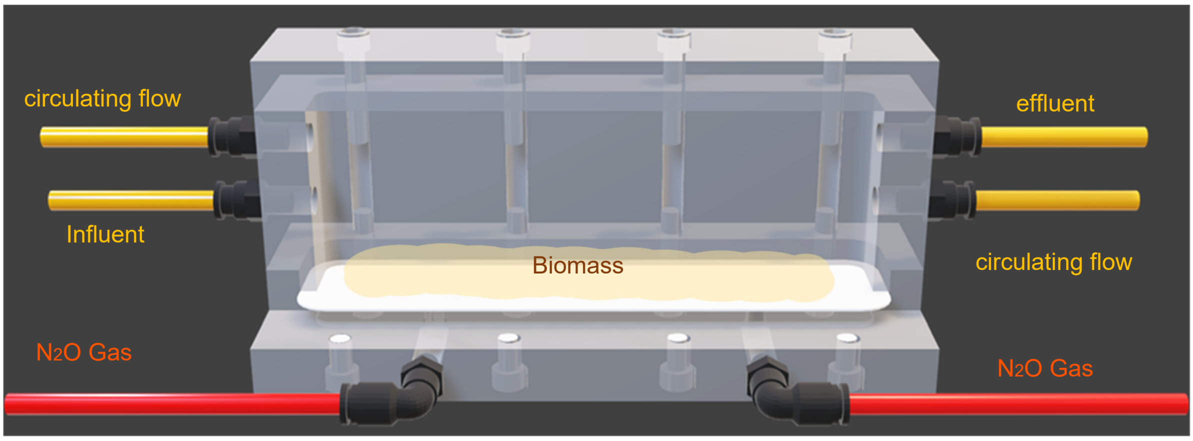

Fig. S1 The schematic illustration of an MBfR used for enriching N_2O-reducing bacteria.

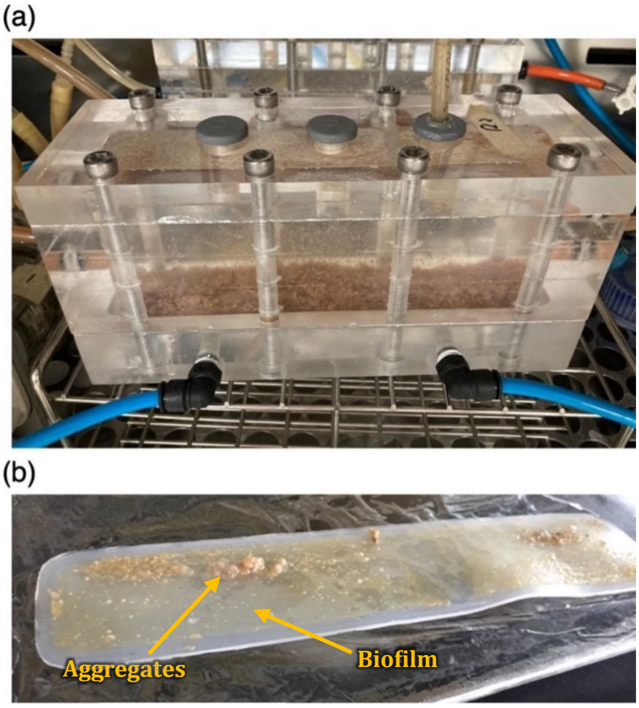

Fig. S2 Biofilm formation onto (a) the side wall of the MBfR and (b) the flat-sheet silicone gas-permeable membrane.

Conditions and probes of FISH

The applied oligonucleotide probes, sequences, and formamide concentrations are shown in **Table S1**. The EUB338mix for all bacteria was a mixture of EUB338, EUB338 II, and EUB338III in equal molar proportions, and the CFX1223/GNSB941 mix for phylum *Chloroflexota* was a mixture of CFX1223 and GNSB941 in equal molar proportions.

Table S1 Primer sequences used in FISH imaging.

Probe name	Sequence (5'-3')	Taxonomy	Formamide [%]	References
AMX368	CCT TTC GGG CAT TGC GAA	*Candidatus Brocadiaceae; Candidatus Brocadiales; Planctomycetia; Planctomycetes; Bacteria*	15	(Schmid *et al.*, 2003)
CFX1223	CCA TTG TAG CGT GTG TGT MG	*Chloroflexota; Bacteria*	35	(Björnsson *et al.*, 2002)
GNSB-941	AAA CCA CAC GCT CCG CT	*Chloroflexota; Bacteria*	35	(Gich *et al.*, 2001)
EUB338	GCT GCC TCC CGT AGG AGT	*Bacteria*	0-50	(Amann *et al.*, 1990)
EUB338 II	GCA GCC ACC CGT AGG TGT	*Planctomycetales; Planctomycetia; Planctomycetes; Bacteria*	0-50	(Daims *et al.*, 1999)
EUB338 III	GCT GCC ACC CGT AGG TGT	*Verrucomicrobiales; Verrucomicrobiae; Verrucomicrobia; Chlamydiae/Verrucomicrobia group; Bacteria*	0-50	(Daims *et al.*, 1999)

R=G/A, Y=T/C, M=A/C, K=G/T, S=G/C, W=A/T, H=A/C/T, B=G/T/C, V=G/C/A, D=G/A/T, N=G/A/T/C

Conditions of quantitative real-time PCR

The applied PCR primer sets and sequences are listed in **Table S2**. The conditions and protocol for quantitative real-time PCR is outlined in the references (Suenaga *et al.*, 2021)

Table S2 Primer sequences used in sequencing and qPCR

Primer name	Target	Sequence (5'-3')	Application	Reference
nirS 4QF	*nirS*	GTSAACGYSAAGGARACSGG	qPCR	(Throback *et al.*, 2004)
nirS 6QR	*nirS*	GASTTCGGRTGSGTCTTSAYGAA	qPCR	(Throback *et al.*, 2004)
nirK876-F	*nirK*	ATYGGCGGVCAYGGCGA	qPCR	(Henry *et al.*, 2004)
nirK1040-R	*nirK*	GCCTCGATCAGRTTRTGGTT	qPCR	(Henry *et al.*, 2004)
qnorB2F	*qnorB*	GGNCAYCARGGNTAYGA	qPCR	(Braker and Tiedje, 2003)
qnorB5R	*qnorB*	ACCCANAGRTGNACNACCCACCA	qPCR	(Braker and Tiedje, 2003)
cnorB2F	*cnorB*	GACAAGNNNTACTGGTGGT	qPCR	(Braker and Tiedje, 2003)
cnorB6R	*cnorB*	GAANCCCCANACNCCNGC	qPCR	(Braker and Tiedje, 2003)
nosZ2F	*nosZ* clade I	CGCRACGGCAASAAGGTSMSSGT	qPCR	(Henry *et al.*, 2006)
nosZ2R	*nosZ* clade I	CAKRTGCAKSGCRTGGCAGAA	qPCR	(Henry *et al.*, 2006)
nosZ-II-F	*nosZ* clade II	CTIGGICCIYTKCAYAC	qPCR	(Jones *et al.*, 2013)
nosZ-II-R	*nosZ* clade II	GCIGARCARAAITCBGTRC	qPCR	(Jones *et al.*, 2013)
515f*	16S rRNA (V4)	AATGATACGGCGACCACCGAGATCTAC ACTATGGTAATTGTGTGCCAGCMGCC GCGGTAA	Amplicon seq.	(Caporaso *et al.*, 2011)
806r**	16S rRNA (V4)	CAAGCAGAAGACGGCATACGAGATXXX XXXXXXXXXAGTCAGTCAGCCGGACTAC HVGGGTWTCTAAT	Amplicon seq.	(Caporaso *et al.*, 2011)

* 5' Illumina adapter, Forward primer pad, Forward primer linker, Forward primer (515f)

** Reverse complement of 3' Illumina adapter, Golay barcode, Reverse primer pad, Reverse primer linker, Reverse primer (806r)

M = A or C; W = A or T; Y = C or T; S = C or G; R = A or G; K= G or T; H = A or C or T; V = A or C or G; B = C or G or T; I = Inosine; X = Tag sequence

Reconstruction and analyses of metagenome-assembled genome

- ***Processing method of high-throughput sequencing of 16S rRNA gene amplicons***

The protocol for library preparation of amplicon sequence is outlined elsewhere (Suenaga *et al.*, 2021). The PCR condition for 16S rRNA gene amplicon sequencing was as follows: initial denaturation at 98°C for 1 min 50 sec, followed by 30 cycles of 98°C for 10 sec, 54°C for 30 sec, 72°C for 30 sec, and then 72°C for 2 min. The PCR reagent consisted of 0.5 µL of Q5 High-Fidelity DNA Polymerase (New England Biolabs, MA), 10 µL of 5 × Q5 reaction buffer (NEB), 1 µL of 10 mM dNTPs (NEB), 2 µL of 10 mM forward and reverse primers, 10 µL of 5 × Q5 high GC enhancer (NEB), 22.5 µL of distilled water and 2 µL of a template. The amplicon was purified with a magnetic bead-based procedure (Agencourt AMPure XP, Beckmancoulter, CA), and the primer dimers were removed by gel cutting and extraction (Wizard® SV Gel and PCR Clean-Up System, Promega, WI). The purified amplicon was quantified by a Qubit fluorometer (Life Technologies, CA) and a Nanodrop 3300 (Thermo Fisher Scientific, MA).

- ***Amplicon sequence analysis***

Sequence data were analyzed using the following bioinformatics tools: Adapter trimming and removal of poor quality base were performed using BBMap/BBduk (v. 38.84) (Bushnell, 2015) with parameters (ktrim=r, k=19, qtrim=r, trimq=8, minlen=36, ftm=5). The trimmed reads were merged using FLASh (version: 2.2.00) (Magoc and Salzberg, 2011) with parameter (m=5) to obtain longer insert length reads. The merged reads into Dada2 (version: 1.26.0) (Prodan *et al.*, 2020) and ASV

clustering was performed. The short insert length reads or low-quality reads in merged reads were removed using Dada2 'FilterAndTirm' function with parameters ("truncQ = 2, minLen=200, maxN = 0, maxEE = 2, rm.phix=TRUE"). Then, error rate estimation (randomize = TRUE), Amplicon Sequence Variant (ASV) inference (default parameters), and chimeras removal (default parameters) were conducted. Separate MiSeq runs were denoised independently and combined after denoising, as required for the generation of accurate error profiles. MultiQC (Ewels *et al.*, 2016) was used to generate integrated reports and check data.

The 16S rRNA ASVs were imported into Qiime2 (Bolyen *et al.*, 2019) (q2cli: 2022.11.1) and taxonomy assignment was performed using QIIME 2's q2-feature-classifier plugin (Bokulich *et al.*, 2018) with pre-trained Silva (138.1) database (Quast *et al.*, 2013; Robeson *et al.*, 2021). Downstream analysis was conducted using R version 4.2.2 (2022-10-31) with ggplot2 package (Wickham, 2016) and phyloseq package (McMurdie and Holmes, 2013). The whole analyses were performed stepwise in a house-made pipeline using Nextflow (Di Tommaso *et al.*, 2017; Ewels *et al.*, 2020).

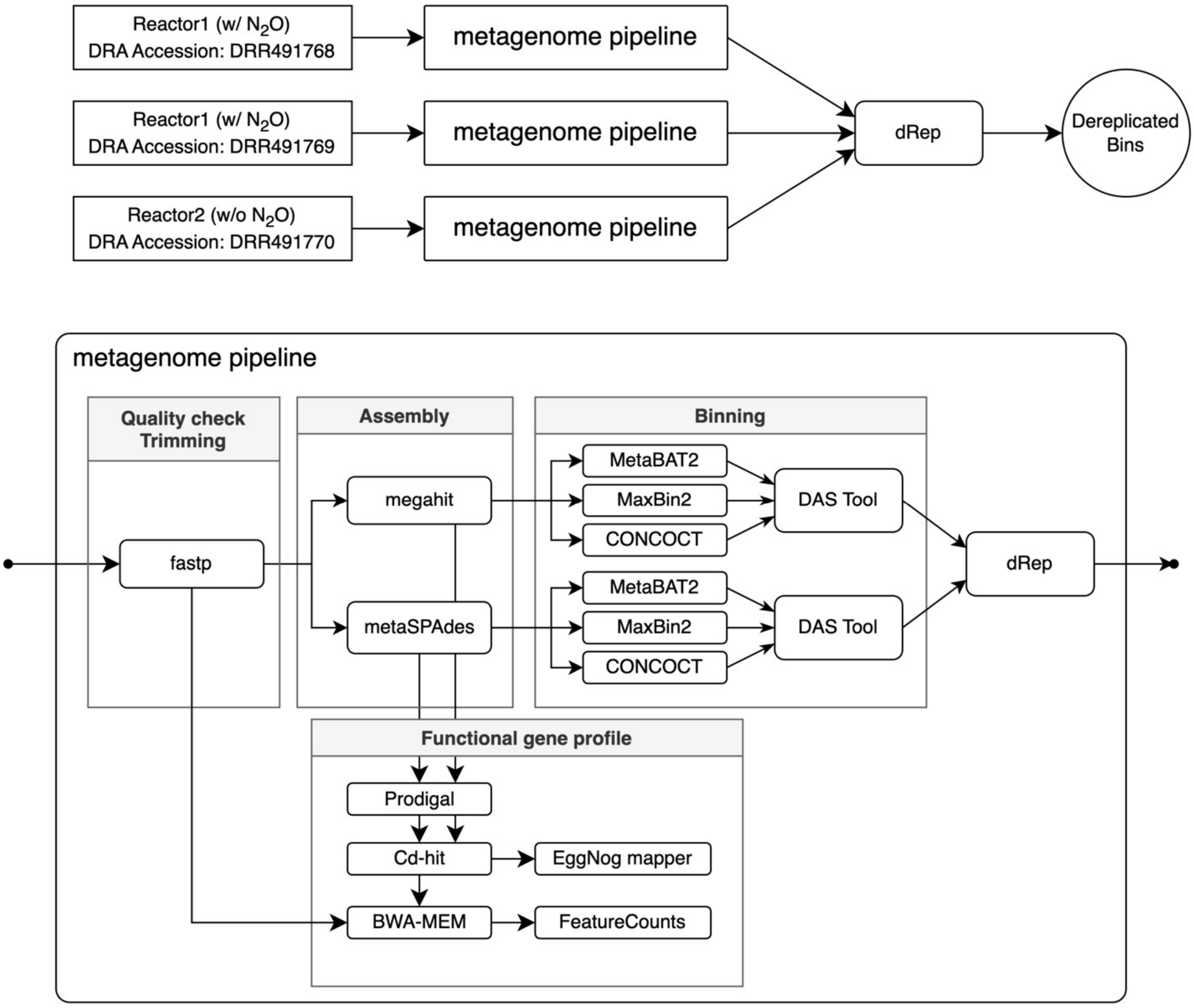

Fig. S3 Overview of reconstruction of metagenome-assembled genomes in this study. The quality of the generated bins was checked using CheckM2 (v1.0.1). Bins (Completeness >= 70 %, Contamination < 5 %) were used for phylogenetic analysis and functional gene annotation.

Functional gene involved in methionine biosynthesis

The methionine biosynthetic pathway in KEGG (Kanehisa and Goto, 2000) is divided into biosynthesis from the precursor uroporphyrinogen to cobyrinate a,c-diamide and from cobyrinate a,c-diamide to cobalamin. The first half of the reaction pathway defines M00924 and M00925 under anaerobic (**Table S3**) and aerobic (**Table S4**) conditions, respectively. The second half, the nucleotide loop assembly pathway, defines the genes associated with M00122 (**Table S5**). These modules were used for the calculation of the fulfillment rate of the methionine synthesis pathway.

Table S3 Genes defined as involved in Cobalamin biosynthesis in anaerobic environments

Gene name	Name	KEGG Orthology	E. C.
cysG	uroporphyrin-III C-methyltransferase / precorrin-2 dehydrogenase	K02302	2.1.1.107 1.3.1.76
cobA	uroporphyrin-III C-methyltransferase	K02303,K13542	2.1.1.107
MET8, sirC	precorrin-2 dehydrogenase	K02304,K24866	1.3.1.76
cbiK, cbiX, cfbA	sirohydrochlorin cobaltochelatase	K02190,K03795,K22011	4.99.1.3
cbiL	cobalt-factor-2 C20-methyltransferase	K03394	2.1.1.151
cbiH	cobalt-factor III methyltransferase	K05934,K13541,K21479	2.1.1.272
cbiF	cobalt-precorrin-4 C11-methyltransferase	K05936	2.1.1.271
cbiG	cobalt-precorrin 5A hydrolase	K02189,K13541	3.7.1.12
cbiD	cobalt-precorrin-5B (C1)-methyltransferase	K02188	2.1.1.195
cbiJ	cobalt-precorrin-6A reductase	K05895	1.3.1.106
cbiT	cobalt-precorrin-6B (C15)-methyltransferase	K02191	2.1.1.196
cbiE	cobalt-precorrin-7 (C5)-methyltransferase	K03399	2.1.1.289
cbiET	cobalt-precorrin-6B C5,C15-methyltransferase	K00595	2.1.1.289 2.1.1.196
cbiC	cobalt-precorrin-8 methylmutase	K06042	5.4.99.60
cbiA	cobyrinic acid a,c-diamide synthase	K02224	6.3.5.11

Table S4 Genes defined as involved in cobalamin biosynthesis in aerobic environments

Gene name	Name	KEGG Orthology	E. C.
cobA	uroporphyrin-III C-methyltransferase	K02303,K13542	2.1.1.107
cobI	precorrin-2 C20-methyltransferase	K03394	2.1.1.130
cobIJ	precorrin-2 C20-methyltransferase / precorrin-3B C17-methyltransferase	K13540	2.1.1.130 2.1.1.131
cobG	precorrin-3B synthase	K02229	1.14.13.83
cobJ	precorrin-3B C17-methyltransferase	K05934,K13541	2.1.1.131
cobM	precorrin-4 C11-methyltransferase	K05936	2.1.1.133
cobF	precorrin-6A synthase	K02228	2.1.1.152
cobK	precorrin-6A reductase	K05895	1.3.1.54
cobL	precorrin-6B C5,15-methyltransferase	K00595	2.1.1.132
cobH	precorrin-8X methylmutase	K06042	5.4.99.61
cobB	cobyrinic acid a,c-diamide synthase	K02224	6.3.5.9
cobNST	cobaltochelatase CobNST	K02230+K09882+K09883	6.6.1.2

Table S5 Genes defined as involved in nucleotide loop assembly

Gene name	Name	KEGG Orthology	E. C.
MMAB, *pduO / cobA, btuR*	cob(I)alamin adenosyltransferase	K00798,K19221	2.5.1.17
cobQ, cbiP	adenosylcobyric acid synthase	K02232	6.3.5.10
cobC1, cobC	cobalamin biosynthetic protein CobC	K02225	RN:R07302 R06529
cbiB, cobD	adenosylcobinamide-phosphate synthase CobD	K02227	6.3.1.10
cobP, cobU	adenosylcobinamide kinase / adenosylcobinamide-phosphate guanylyltransferase	K02231	2.7.1.156 2.7.7.62
cobU, cobT	nicotinate-nucleotide--dimethylbenzimidazole phosphoribosyltransferase	K00768	2.4.2.21
cobC, phpB	alpha-ribazole phosphatase	K02226	3.1.3.73
rhnA-cobC	adenosylcobalamin/alpha-ribazole phosphatase	K22316	3.1.26.4 3.1.3.73
cobS, cobV	adenosylcobinamide-GDP ribazoletransferase	K02233	2.7.8.26

Peptidoglycan degradation

The genes in **Table S6** were used to calculate the fulfillment rate of the peptidoglycan degradation and recycling pathway based on the amino sugar and nucleotide sugar metabolism (map00520) in KEGG (Kanehisa and Goto, 2000).

Table S6 Genes defined as involved in peptidoglycan degradation

Gene name	Name	KEGG Orthology	E. C.
nagZ	β-*N*-Acetylglucosaminidase	K01207	2.7.1.59
nagK	*N*-Acetylglucosamine Kinase	K00884	2.7.1.59
nagE	N-acetyl-D-glucosamine PTS permease	K02804	2.7.1.193
nagA	N-Acetylglucosamine-6-P Deacetylase	K01443	3.5.1.25
nagB	glucosamine-6-phosphate deaminase	K02564	3.5.99.6
murP	N-acetylmuramic acid PTS system EIICB component	K11192	2.7.1.192
AnmK	Anhydro-N-Acetylmuramic Acid Kinase	K09001	2.7.1.170
murQ	N-Acetylmuramic Acid-6-P Etherase	K07106	4.2.1.126

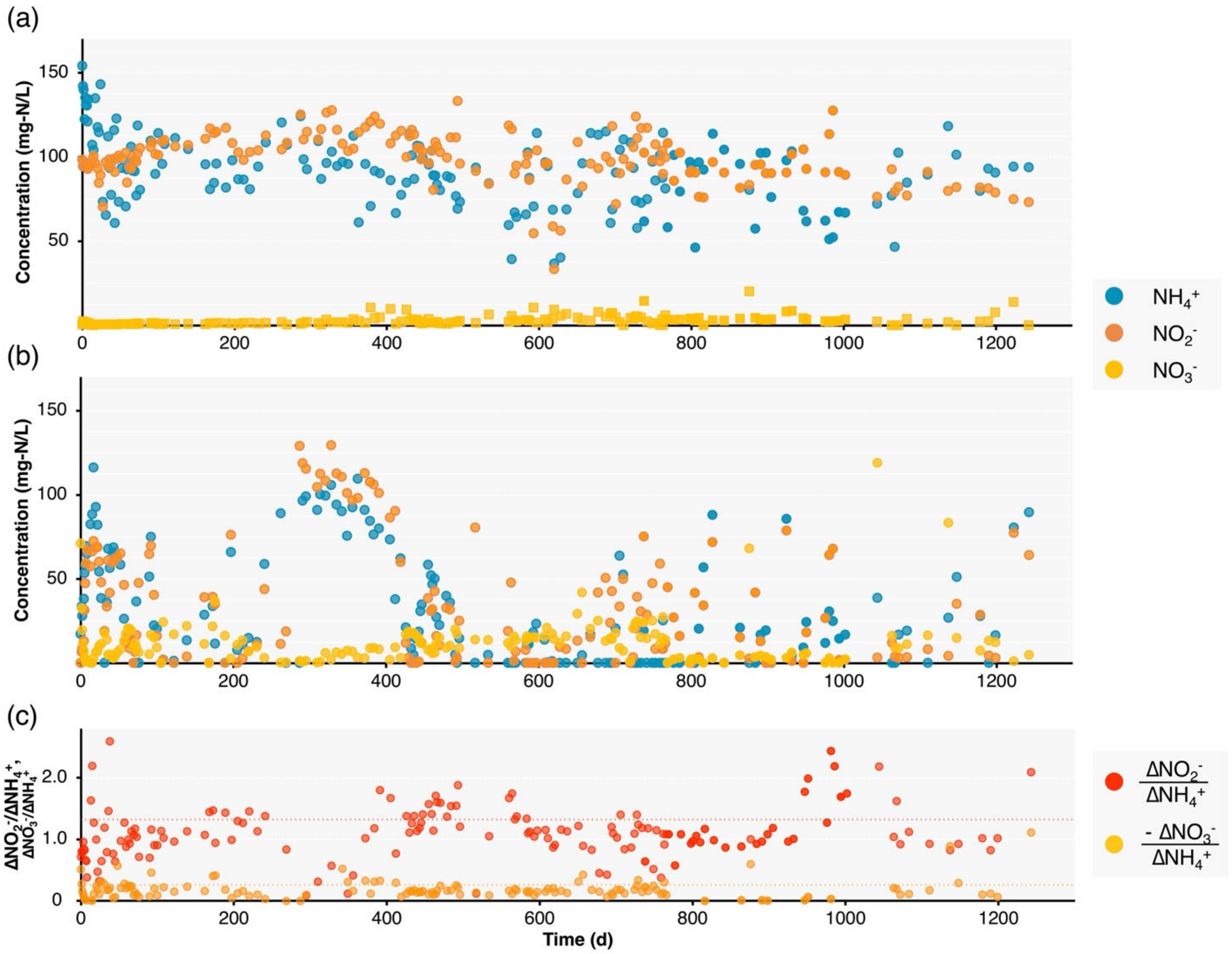

Fig. S4 Time courses of nitrogen concentrations in (a) influent and (b) effluent of Reactor 1. In Reactor 1, NH_4^+ and NO_2^- with 100 mg-N/L each were supplied with N_2O supply through the gas permeable membrane. (c) The stoichiometric ratio of NO_2^- consumption over NH_4^+ consumption and NO_3^- production over NH_4^+ consumption. The dashed lines represent these stoichiometric ratios by anammox reaction (Strous *et al.* 1998).

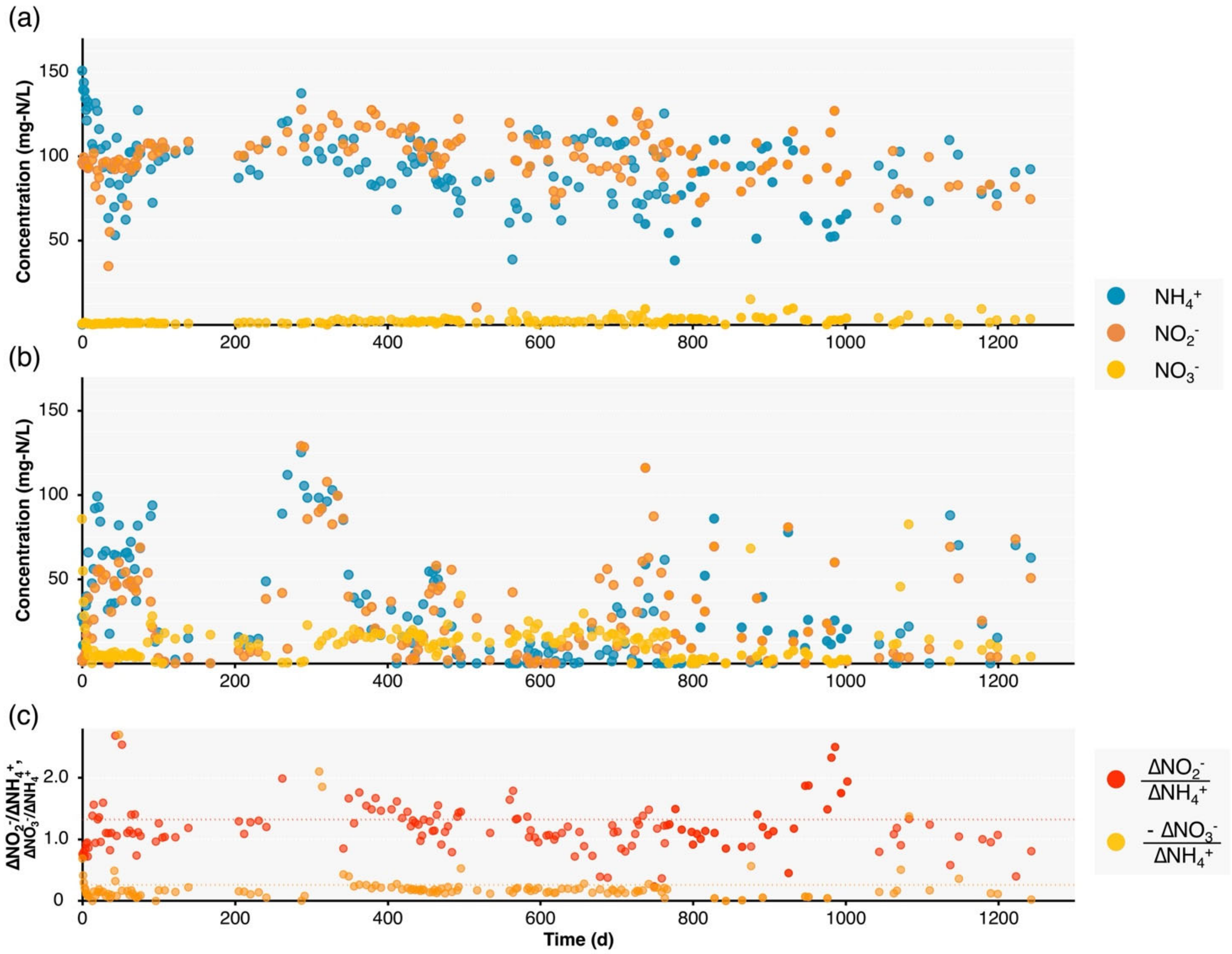

Fig. S5 Timem courses of nitrogen concentrations in (a) influent and (b) effluent of Reactor 2. In Reactor 2, NH_4^+ and NO_2^- with 100 mg-N/L each were supplied. (c) The stoichiometric ratio of NO_2^- consumption over NH_4^+ consumption and NO_3^- production over NH_4^+ consumption. The dashed lines represent these stoichiometric ratios by anammox reaction (Strous *et al.* 1998).

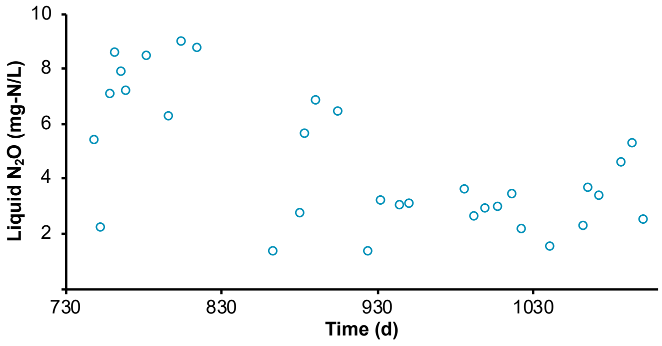

Fig. S6 Dissolved N_2O concentration in Reactor 1 (N_2O was supplied)

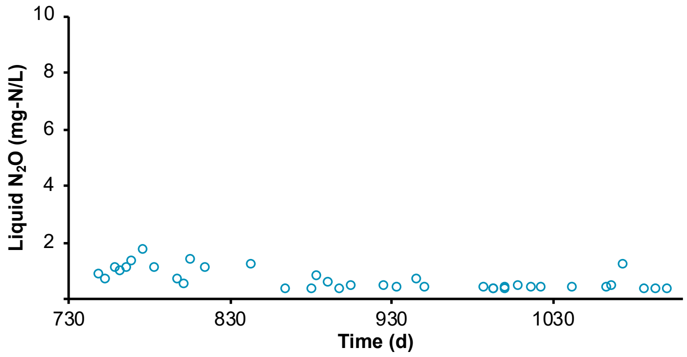

Fig. S7 Dissolved N_2O concentration in Reactor 2, where N_2O was not supplied.

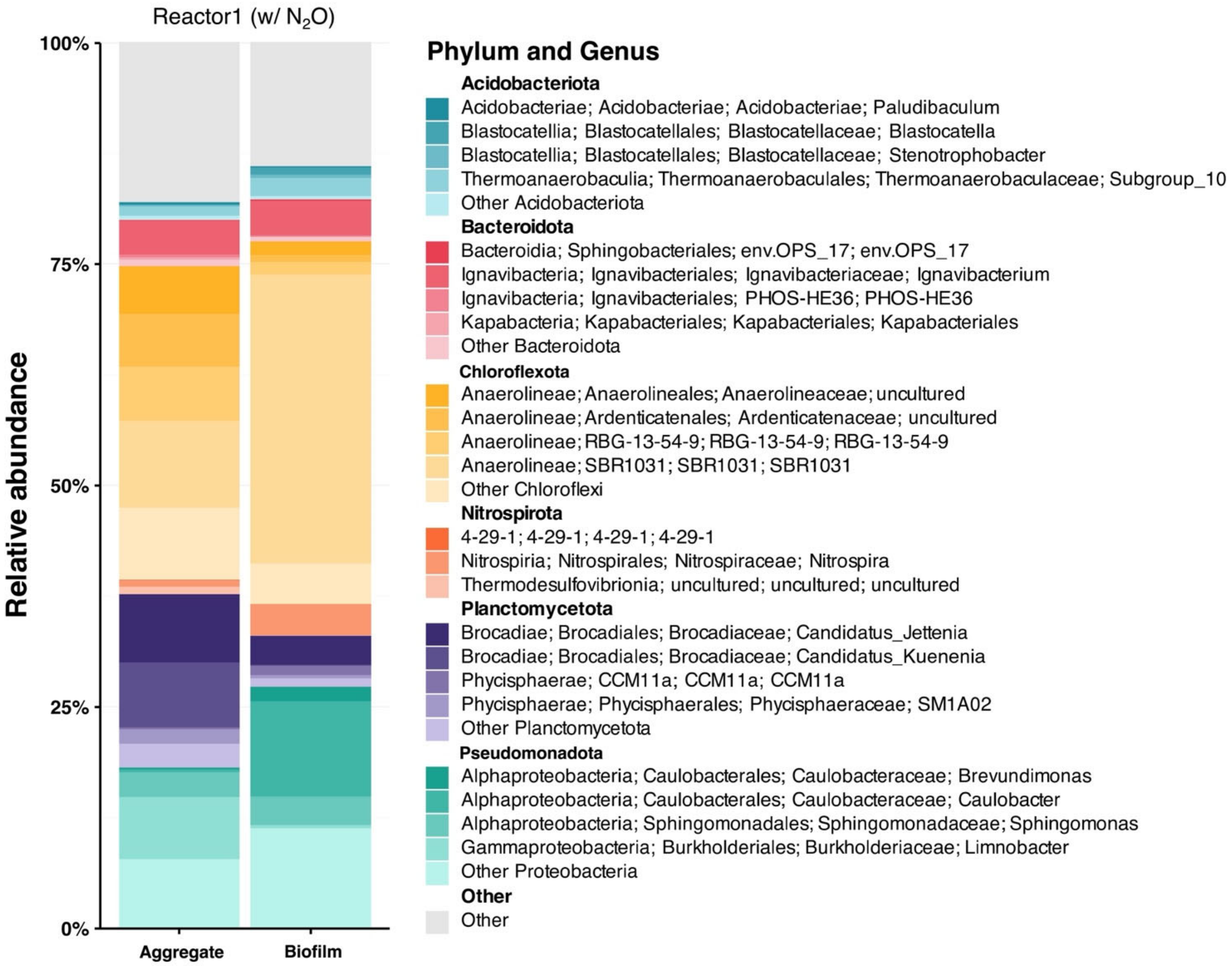

Fig. S8 Microbial community compositions of the dense aggregates (aggregate), deposited onto the gas-permeable membrane, and the biofilm grown on the membrane (biofilm) by the 16S rRNA gene amplicons in Reactor 1 (w/ N_2O supply) on day 199. The top six phyla and genera during the overall incubation are listed.

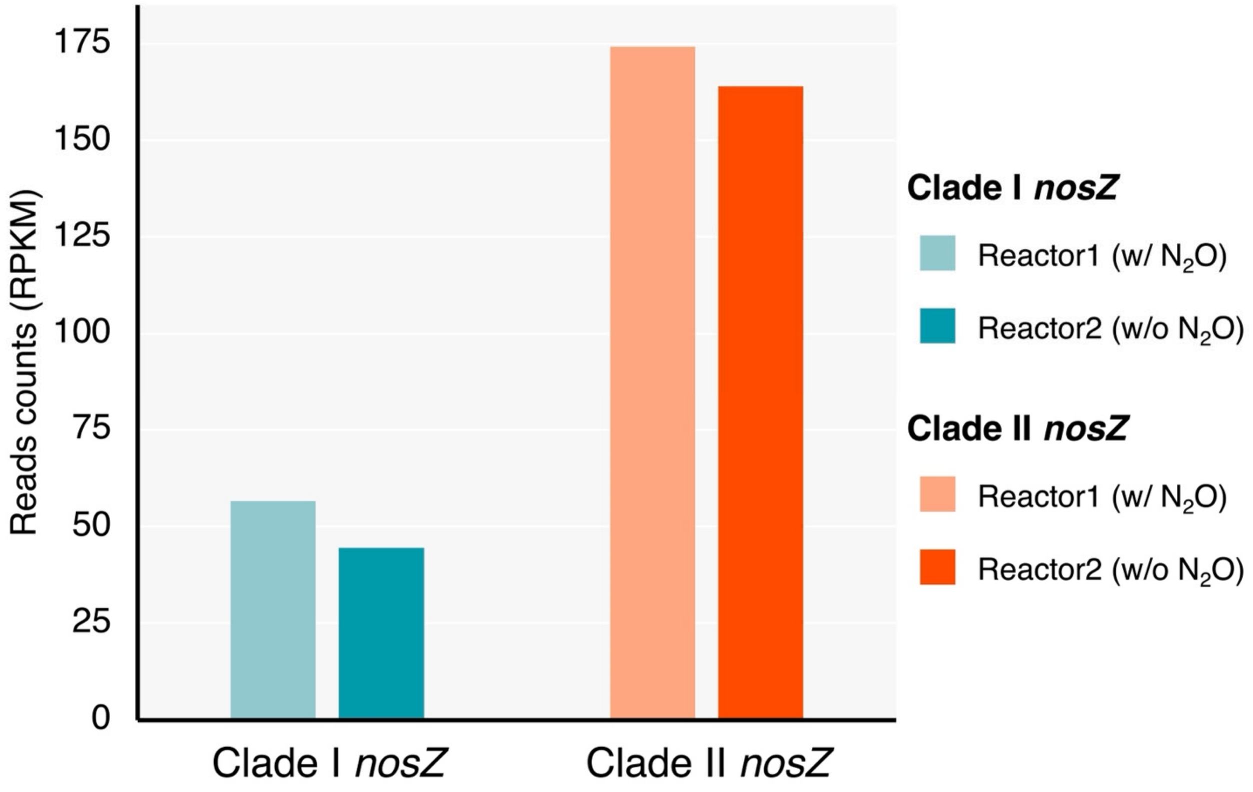

Fig. S9 Normalized read counts of Clade I *nosZ* and Clade II *nosZ*-coding sequences in the assembled contigs. In both genes, the lighter and darker colors indicate samples taken from Reactor 1 and Reactor 2, respectively.

Table S7 DDBJ accession numbers of the metagenome-assembled genomes (MAGs)

Bin	Accession numbers
bin12	BTUB01000001-BTUB01000194
bin13	BTUC01000001-BTUC01000665
bin16	BTUD01000001-BTUD01000097
bin17	BTUE01000001-BTUE01000079
bin19	BTUF01000001-BTUF01000024
bin20	BTUG01000001-BTUG01000092
bin22	BTUH01000001-BTUH01000080
bin23	BTUI01000001-BTUI01000030
bin25	BTUJ01000001-BTUJ01000096
bin26	BTUK01000001-BTUK01000157
bin31	BTUL01000001-BTUL01000064
bin33	BTUM01000001-BTUM01000166
bin34	BTUN01000001-BTUN01000725
bin36	BTUO01000001-BTUO01000017
bin37	BTUP01000001-BTUP01000040
bin42	BTUQ01000001-BTUQ01000269
bin44	BTUR01000001-BTUR01000059
bin45	BTUS01000001-BTUS01000018
bin46	BTUT01000001-BTUT01000910
bin47	BTUU01000001-BTUU01000042
bin48	BTUV01000001-BTUV01000191
bin49	BTUW01000001-BTUW01000064
bin4	BTUX01000001-BTUX01000442
bin50	BTUY01000001-BTUY01000211
bin51	BTUZ01000001-BTUZ01000162
bin52	BTVA01000001-BTVA01000240
bin53	BTVB01000001-BTVB01000085
bin55	BTVC01000001-BTVC01000143
bin56	BTVD01000001-BTVD01000161
bin57	BTVE01000001-BTVE01000180
bin58	BTVF01000001-BTVF01000076
bin59	BTVG01000001-BTVG01000106
bin60	BTVH01000001-BTVH01000089

bin61	BTVI01000001-BTVI01000094
bin64	BTVJ01000001-BTVJ01000186
bin66	BTVK01000001-BTVK01000200
bin67	BTVL01000001-BTVL01000108
bin68	BTVM01000001-BTVM01000066
bin6	BTVN01000001-BTVN01000064
bin72	BTVO01000001-BTVO01000188
bin74	BTVP01000001-BTVP01000086
bin75	BTVQ01000001-BTVQ01000111
bin76	BTVR01000001-BTVR01000031
bin77	BTVS01000001-BTVS01000043
bin78	BTVT01000001-BTVT01000080
bin79	BTVU01000001-BTVU01000141
bin7	BTVV01000001-BTVV01000287
bin80	BTVW01000001-BTVW01000069
bin81	BTVX01000001-BTVX01000049
bin82	BTVY01000001-BTVY01000105
bin83	BTVZ01000001-BTVZ01000622
bin84	BTWA01000001-BTWA01000088
bin85	BTWB01000001-BTWB01000022
bin8	BTWC01000001-BTWC01000084

Table S8 Quality information on the retrieved MAGs

	Genome size (bp)	GC (%)	# CDS	Completeness (%)	Contamination (%)	# contigs	N50 (bp)	16S/23S/5S rRNA (nt)	# tRNA / # tRNA_types
bin10	3,175,465	53.8	3,939	74.04	2.59	1,397	2673	n.d./n.d./109	30/17
bin12	1,218,947	37.6	1,362	90.19	3.19	211	10994	400/n.d./108	30/20
bin13	7,332,032	63.8	6,076	92.28	4.9	763	13590	1112/n.d./79	41/20
bin16	1,475,422	54.2	1,513	98.05	1.07	100	778725	1415/1222/76	56/21
bin17	3,727,900	39.9	3,294	99.7	1.08	85	71645	n.d./n.d./n.d.	44/21
bin19	2,726,436	61.1	2,499	98.46	0.07	38	126913	1497/2927/108	48/23
bin20	4,674,782	61	3,765	95.83	1.34	103	79110	1227/2878/76	46/23
bin22	4,295,676	65.6	3,385	97.62	1	80	161311	n.d./1210/82	46/22
bin23	2,898,334	70.9	2,867	95.06	3.44	30	228525	1460/2908/79	51/23
bin25	3,831,034	68.4	3,303	98.9	0.71	96	102230	n.d./n.d./102	44/21
bin26	2,838,702	67.4	2,774	99.18	0.32	157	24495	n.d./n.d./110	36/20
bin30	3,001,080	33.8	3,960	74.49	4.92	1,395	2297	n.d./n.d./n.d.	46/19
bin31	3,133,417	66.8	3,111	96.39	0.29	64	84818	n.d./n.d./n.d.	42/21
bin33	8,359,614	65.8	7,460	97.59	2.64	166	79372	n.d./n.d./n.d.	60/23
bin34	6,835,540	70.7	6,510	94.98	1.22	725	13368	n.d./n.d./110	47/22
bin36	4,561,886	71.8	3,892	98.41	1.43	24	356768	623/2970/109	47/23
bin37	3,084,571	69.3	2,888	93.28	0.1	44	121915	n.d./n.d./n.d.	35/18
bin4	4,736,018	60.7	4,152	95.71	2.24	442	15503	n.d./n.d./n.d.	39/20
bin42	3,695,611	70.9	3,601	91.82	1.21	331	15361	n.d./n.d./n.d.	30/15
bin44	734,039	51.9	793	98.17	0.55	64	115902	725/1480/70	49/20
bin45	2,911,803	68.1	2,679	100	0.03	20	208602	800/2865/110	47/22
bin46	3,872,042	72.2	4,357	91.67	2.6	961	5875	n.d./1903/109	39/20
bin47	5,659,677	66.1	4,610	96.23	1.83	50	217992	482/1862/79	56/24
bin48	3,856,622	42.7	3,115	99.5	0.15	239	26899	816/n.d./n.d.	46/21
bin49	3,992,923	47.4	3,237	93.97	0.39	89	113260	n.d./1810/111	44/21
bin5	4,928,293	64.2	5,198	73.02	2.72	1,082	5411	n.d./n.d./n.d.	43/20
bin50	3,429,644	34.5	3,060	99.97	1.89	225	29837	n.d./1008/111	35/17
bin51	3,693,672	34.8	3,233	92.69	0.41	209	30963	n.d./n.d./n.d.	42/21
bin52	3,723,666	68.9	3,710	92.18	2.65	253	31807	n.d./n.d./n.d.	40/19
bin53	5,165,364	71.6	4,564	96.03	3.4	107	64582	818/n.d./n.d.	51/23
bin55	4,281,804	70.5	3,640	98.5	1.04	143	44636	n.d./n.d./98	47/21
bin56	9,189,370	69.9	8,365	99.35	0.81	161	112205	1543/4029/108	69/24
bin57	4,524,068	65.2	4,132	100	2.29	180	40606	1540/2957/109	48/23
bin58	4,866,481	67.7	3,953	91.73	1.25	76	196629	1466/2433/100	51/22
bin59	5,255,173	66.4	4,375	92.1	2.75	106	153284	852/n.d./80	44/19
bin6	2,907,508	64.6	2,753	98.44	1.35	64	74083	n.d./n.d./n.d.	38/20
bin60	3,705,566	56.6	3,330	98.42	1.01	89	65244	1464/3158/110	46/22
bin61	3,362,841	62.7	2,951	97.31	0.28	94	74557	1509/2871/108	49/23
bin64	8,772,155	70.9	6,422	97.39	3.45	186	90600	n.d./n.d./n.d.	47/22
bin66	4,452,073	72.2	4,097	93.09	1.53	200	35413	n.d./n.d./106	41/19
bin67	4,028,106	60.3	3,899	99.95	3.35	108	68319	797/n.d./109	54/21
bin68	2,529,118	49.7	2,100	100	0.18	66	58360	n.d./n.d./n.d.	38/20
bin7	6,933,721	63.8	5,631	94.78	1.55	287	43155	929/n.d./n.d.	79/22
bin72	4,250,605	69.1	4,174	93.71	2.63	300	26648	682/n.d./108	44/21
bin74	3,508,827	63.3	3,376	99.94	1.16	94	63827	n.d./n.d./n.d.	44/20
bin75	4,385,503	54.5	3,452	94.22	0.63	173	52061	960/2631/69	48/22
bin76	2,680,838	66.2	2,585	99.61	0	33	624822	798/948/110	44/20
bin77	3,335,517	68.9	2,892	97.61	1.13	49	251559	1478/1098/n.d.	46/22

bin78	4,095,259	71.3	3,880	96.45	0.44	89	98903	n.d./n.d./n.d.	41/21
bin79	2,357,215	30.7	2,125	100	0.04	157	24694	450/2770/103	34/21
bin8	3,479,271	66.6	3,149	100	3.63	102	59803	n.d./n.d./110	43/17
bin80	2,894,152	67.7	2,977	100	0.38	97	47004	764/n.d./110	47/20
bin81	3,432,083	64.5	3,191	99.78	0.66	66	93905	1500/2920/108	52/23
bin82	6,754,206	60	5,450	99.39	0.94	125	98008	723/1258/110	47/21
bin83	4,733,208	68.2	4,017	91.95	4.81	676	9589	n.d./n.d./n.d.	34/19
bin84	6,335,068	56.6	5,187	96.25	1.89	102	119009	717/948/110	50/21
bin85	1,178,540	49.1	1,223	97.99	0.05	36	78057	n.d./2917/102	42/20

Table S9 Taxonomy assignment using GTDB-Tk with GTDB (r207) database

bin	Taxonomy
bin10	d__Bacteria;p__Acidobacteriota;c__Blastocatellia;o__Pyrinomonadales;f__Pyrinomonadaceae;g__OLB17;s__
bin12	d__Bacteria;p__Dependentiae;c__Babeliae;o__Babeliales;f__Vermiphilaceae;g__;s__
bin13	d__Bacteria;p__Planctomycetota;c__Phycisphaerae;o__UBA1845;f__PWPN01;g__JAAXZI01;s__
bin16	d__Bacteria;p__Patescibacteria;c__ABY1;o__SG8-24;f__2-12-FULL-60-25;g__2-12-FULL-60-25;s__2-12-FULL-60-25 sp003576745
bin17	d__Bacteria;p__Planctomycetota;c__Brocadiae;o__Brocadiales;f__Brocadiaceae;g__Jettenia;s__Jettenia caeni
bin19	d__Bacteria;p__Armatimonadota;c__Fimbriimonadia;o__Fimbriimonadales;f__Fimbriimonadaceae;g__H1-ARM1;s__H1-ARM1 sp001567425
bin20	d__Bacteria;p__Planctomycetota;c__Phycisphaerae;o__UBA1845;f__UTPLA1;g__UTPLA1;s__UTPLA1 sp002050205
bin22	d__Bacteria;p__Planctomycetota;c__Phycisphaerae;o__UBA1845;f__UBA1845;g__JABWBH01;s__JABWBH01 sp013360585
bin23	d__Bacteria;p__Chloroflexota;c__Dehalococcoidia;o__UBA2979;f__UBA2979;g__W-Chloroflexi-9;s__
bin25	d__Bacteria;p__Proteobacteria;c__Gammaproteobacteria;o__Xanthomonadales;f__SZUA-5;g__JADKFD01;s__JADKFD01 sp019187485
bin26	d__Bacteria;p__Proteobacteria;c__Gammaproteobacteria;o__Pseudomonadales;f__Porticoccaceae;g__JAGPUQ01;s__
bin30	d__Bacteria;p__Firmicutes_A;c__Clostridia;o__Eubacteriales;f__Alkalibacteraceae;g__M08DMB;s__
bin31	d__Bacteria;p__Proteobacteria;c__Gammaproteobacteria;o__Burkholderiales;f__Rhodocyclaceae;g__Desulfobacillus;s__Desulfobacillus denitrificans
bin33	d__Bacteria;p__Eremiobacterota;c__Xenobia;o__Xenobiales;f__JADMJV01;g__;s__
bin34	d__Bacteria;p__Myxococcota;c__Myxococcia;o__Myxococcales;f__Myxococcaceae;g__;s__
bin36	d__Bacteria;p__Acidobacteriota;c__Thermoanaerobaculia;o__UBA5704;f__UBA5704;g__JACTMI01;s__
bin37	d__Bacteria;p__Proteobacteria;c__Gammaproteobacteria;o__Steroidobacterales;f__Steroidobacteraceae;g__CADEFQ01;s__
bin4	d__Bacteria;p__Hydrogenedentota;c__Hydrogenedentia;o__Hydrogenedentiales;f__SLHB01;g__JABWCD01;s__
bin42	d__Bacteria;p__Proteobacteria;c__Gammaproteobacteria;o__Burkholderiales;f__Casimicrobiaceae;g__VBCG01;s__VBCG01 sp015075645
bin44	d__Bacteria;p__Patescibacteria;c__Paceibacteria;o__UBA9983_A;f__EsbW-18;g__EsbW-18;s__
bin45	d__Bacteria;p__Proteobacteria;c__Gammaproteobacteria;o__GCA-2729495;f__GCA-2729495;g__QUBU01;s__QUBU01 sp014337915
bin46	d__Bacteria;p__Actinobacteriota;c__Acidimicrobiia;o__IMCC26256;f__PALSA-555;g__CAIUKV01;s__
bin47	d__Bacteria;p__Planctomycetota;c__Phycisphaerae;o__UBA1845;f__Fen-1342;g__JACRLV01;s__
bin48	d__Bacteria;p__Bacteroidota;c__Ignavibacteria;o__Ignavibacteriales;f__Ignavibacteriaceae;g__H2-BAC3;s__
bin49	d__Bacteria;p__Bacteroidota;c__Ignavibacteria;o__Ignavibacteriales;f__Ignavibacteriaceae;g__UTCHB3;s__UTCHB3 sp008363265
bin5	d__Bacteria;p__Acidobacteriota;c__Acidobacteriae;o__Bryobacterales;f__Bryobacteraceae;g__PNKE01;s__
bin50	d__Bacteria;p__Bacteroidota;c__Ignavibacteria;o__Ignavibacteriales;f__Ignavibacteriaceae;g__Ignavibacterium;s__Ignavibacterium album_C
bin51	d__Bacteria;p__Bacteroidota;c__Ignavibacteria;o__Ignavibacteriales;f__Ignavibacteriaceae;g__IGN2;s__IGN2 sp013285405
bin52	d__Bacteria;p__Proteobacteria;c__Gammaproteobacteria;o__Burkholderiales;f__Burkholderiaceae;g__SCN-69-89;s__
bin53	d__Bacteria;p__Gemmatimonadota;c__Gemmatimonadetes;o__Longimicrobiales;f__UBA6960;g__SZUA-318;s__
bin55	d__Bacteria;p__Gemmatimonadota;c__Gemmatimonadetes;o__Gemmatimonadales;f__Gemmatimonadaceae;g__SCN-70-22;s__SCN-70-22 sp001724275
bin56	d__Bacteria;p__Myxococcota;c__Polyangia;o__Polyangiales;f__Polyangiaceae;g__JADJKB01;s__JADJKB01 sp015075635
bin57	d__Bacteria;p__Acidobacteriota;c__Vicinamibacteria;o__Vicinamibacterales;f__SCN-69-37;g__SYFT01;s__
bin58	d__Bacteria;p__Planctomycetota;c__Phycisphaerae;o__Phycisphaerales;f__UBA1924;g__JAEUJB01;s__
bin59	d__Bacteria;p__Proteobacteria;c__Gammaproteobacteria;o__Xanthomonadales;f__Rhodanobacteraceae;g__Tahibacter;s__
bin6	d__Bacteria;p__Proteobacteria;c__Gammaproteobacteria;o__Burkholderiales;f__Rhodocyclaceae;g__CG2-30-68-42;s__
bin60	d__Bacteria;p__Chloroflexota;c__Anaerolineae;o__Anaerolineales;f__EnvOPS12;g__OLB14;s__OLB14 sp900696595
bin61	d__Bacteria;p__Armatimonadota;c__Fimbriimonadia;o__Fimbriimonadales;f__;g__;s__
bin64	d__Bacteria;p__Myxococcota;c__UBA9042;o__JABWCM01;f__;g__;s__
bin66	d__Bacteria;p__Proteobacteria;c__Gammaproteobacteria;o__Burkholderiales;f__Burkholderiaceae;g__Rubrivivax;s__
bin67	d__Bacteria;p__Nitrospirota;c__Nitrospiria;o__Nitrospirales;f__Nitrospiraceae;g__Nitrospira_A;s__Nitrospira_A sp001567445
bin68	d__Bacteria;p__Bacteroidota;c__Kapabacteria;o__Kapabacteriales;f__Kapabacteriaceae;g__OLB6;s__OLB6 sp001567175
bin7	d__Bacteria;p__Planctomycetota;c__SZUA-567;o__H5-PLA8;f__H5-PLA8;g__H5-PLA8;s__
bin72	d__Bacteria;p__Proteobacteria;c__Gammaproteobacteria;o__Burkholderiales;f__Burkholderiaceae;g__DSNY01;s__DSNY01 sp016861185
bin74	d__Bacteria;p__Proteobacteria;c__Alphaproteobacteria;o__Parvibaculales;f__Parvibaculaceae;g__Parvibaculum;s__
bin75	d__Bacteria;p__OLB16;c__OLB16;o__OLB16;f__OLB16;g__OLB16;s__OLB16 sp001567115
bin76	d__Bacteria;p__Proteobacteria;c__Gammaproteobacteria;o__UBA5335;f__UBA5335;g__Macondimonas;s__

bin77	d__Bacteria;p__Planctomycetota;c__Phycisphaerae;o__Phycisphaerales;f__UBA1924;g__CAADGN01;s__CAADGN01 sp900696545
bin78	d__Bacteria;p__Proteobacteria;c__Gammaproteobacteria;o__Burkholderiales;f__Burkholderiaceae;g__Rubrivivax;s__
bin79	d__Bacteria;p__Bacteroidota;c__Bacteroidia;o__Chitinophagales;f__Chitinophagaceae;g__UBA1930;s__
bin8	d__Bacteria;p__Proteobacteria;c__Gammaproteobacteria;o__GCA-2729495;f__GCA-2729495;g__QUBU01;s__QUBU01 sp011526045
bin80	d__Bacteria;p__Chloroflexota;c__Dehalococcoidia;o__Tepidiformales;f__Tepidiformaceae;g__FeB-14;s__
bin81	d__Bacteria;p__Armatimonadota;c__Fimbriimonadia;o__Fimbriimonadales;f__Fimbriimonadaceae;g__UBA2387;s__
bin82	d__Bacteria;p__Hydrogenedentota;c__Hydrogenedentia;o__Hydrogenedentiales;f__SLHB01;g__JABWCD01;s__
bin83	d__Bacteria;p__Planctomycetota;c__Phycisphaerae;o__UBA1845;f__PWPN01;g__JAAXZI01;s__
bin84	d__Bacteria;p__Hydrogenedentota;c__Hydrogenedentia;o__Hydrogenedentiales;f__SLHB01;g__;s__
bin85	d__Bacteria;p__Chloroflexota;c__Dehalococcoidia;o__Dehalococcoidales;f__Dehalococcoidaceae;g__Dehalococcoides;s__Dehalococcoides mccartyi

References

Amann, R.I., Binder, B.J., Olson, R.J., Chisholm, S.W., Devereux, R. and Stahl, D.A. (1990) Combination of 16S rRNA-targeted oligonucleotide probes with flow cytometry for analyzing mixed microbial populations. *Appl Environ Microbiol* **56**: 1919-1925.

Björnsson, L., Hugenholtz, P., Tyson, G.W. and Blackall, L.L. (2002) Filamentous Chloroflexi (green non-sulfur bacteria) are abundant in wastewater treatment processes with biological nutrient removal. *Microbiology (Reading)* **148**: 2309-2318.

Bokulich, N.A., Kaehler, B.D., Rideout, J.R., Dillon, M., Bolyen, E., Knight, R*., et al.* (2018) Optimizing taxonomic classification of marker-gene amplicon sequences with QIIME 2's q2-feature-classifier plugin. *Microbiome* **6**: 90.

Bolyen, E., Rideout, J.R., Dillon, M.R., Bokulich, N.A., Abnet, C.C., Al-Ghalith, G.A*., et al.* (2019) Reproducible, interactive, scalable and extensible microbiome data science using QIIME 2. *Nat Biotechnol* **37**: 852-857.

Braker, G. and Tiedje, J.M. (2003) Nitric oxide reductase (norB) genes from pure cultures and environmental samples. *Appl Environ Microbiol* **69**: 3476-3483.

Bushnell B. (2015). BBMap. URL http://www.epa.gov/gmpo/about/facts.html

Caporaso, J.G., Lauber, C.L., Walters, W.A., Berg-Lyons, D., Lozupone, C.A., Turnbaugh, P.J*., et al.* (2011) Global patterns of 16S rRNA diversity at a depth of millions of sequences per sample. *Proc Natl Acad Sci U S A* **108 Suppl 1**: 4516-4522.

Daims, H., Brühl, A., Amann, R., Schleifer, K.H. and Wagner, M. (1999) The domain-specific probe EUB338 is insufficient for the detection of all Bacteria: development and evaluation of a more comprehensive probe set. *Syst Appl Microbiol* **22**: 434-444.

Di Tommaso, P., Chatzou, M., Floden, E.W., Barja, P.P., Palumbo, E. and Notredame, C. (2017) Nextflow enables reproducible computational workflows. *Nat Biotechnol* **35**: 316-319.

Ewels, P., Magnusson, M., Lundin, S. and Kaller, M. (2016) MultiQC: summarize analysis results for multiple tools and samples in a single report. *Bioinformatics* **32**: 3047-3048.

Ewels, P.A., Peltzer, A., Fillinger, S., Patel, H., Alneberg, J., Wilm, A*., et al.* (2020) The nf-core framework for community-curated bioinformatics pipelines. *Nat Biotechnol* **38**: 276-278.

Gich, F., Garcia-Gil, J. and Overmann, J. (2001) Previously unknown and phylogenetically diverse members of the green nonsulfur bacteria are indigenous to freshwater lakes. *Arch Microbiol* **177**: 1-10.

Henry, S., Baudoin, E., Lopez-Gutierrez, J.C., Martin-Laurent, F., Brauman, A. and Philippot, L. (2004) Quantification of denitrifying bacteria in soils by nirK gene targeted real-time PCR. *J Microbiol Methods* **59**: 327-335.

Henry, S., Bru, D., Stres, B., Hallet, S. and Philippot, L. (2006) Quantitative detection of the nosZ gene, encoding nitrous oxide reductase, and comparison of the abundances of 16S rRNA, narG, nirK, and nosZ genes in soils. *Appl Environ Microbiol* **72**: 5181-5189.

Jones, C.M., Graf, D.R., Bru, D., Philippot, L. and Hallin, S. (2013) The unaccounted yet abundant nitrous oxide-reducing microbial community: a potential nitrous oxide sink. *ISME J* **7**: 417-426.

Kanehisa, M. and Goto, S. (2000) KEGG: kyoto encyclopedia of genes and genomes. *Nucleic Acids Res* **28**: 27-30.

Magoc, T. and Salzberg, S.L. (2011) FLASH: fast length adjustment of short reads to improve genome assemblies. *Bioinformatics* **27**: 2957-2963.

McMurdie, P.J. and Holmes, S. (2013) phyloseq: an R package for reproducible interactive analysis and graphics of microbiome census data. *PLoS One* **8**: e61217.

Prodan, A., Tremaroli, V., Brolin, H., Zwinderman, A.H., Nieuwdorp, M. and Levin, E. (2020) Comparing bioinformatic pipelines for microbial 16S rRNA amplicon sequencing. *PLoS One* **15**: e0227434.

Quast, C., Pruesse, E., Yilmaz, P., Gerken, J., Schweer, T., Yarza, P.*, et al.* (2013) The SILVA ribosomal RNA gene database project: improved data processing and web-based tools. *Nucleic Acids Res* **41**: D590-596.

Robeson, M.S., 2nd, O'Rourke, D.R., Kaehler, B.D., Ziemski, M., Dillon, M.R., Foster, J.T. and Bokulich, N.A. (2021) RESCRIPt: Reproducible sequence taxonomy reference database management. *PLoS Comput Biol* **17**: e1009581.

Schmid, M., Walsh, K., Webb, R., Rijpstra, W.I., van de Pas-Schoonen, K., Verbruggen, M.J.*, et al.* (2003) Candidatus "Scalindua brodae", sp. nov., Candidatus "Scalindua wagneri", sp. nov., two new species of anaerobic ammonium oxidizing bacteria. *Syst Appl Microbiol* **26**: 529-538.

Strous, M., Heijnen, J.J., Kuenen, J.G. and Jetten, M.S.M. (1998) The sequencing batch reactor as a powerful tool for the study of slowly growing anaerobic ammonium-oxidizing microorganisms. *Appl Microbiol Biotechnol* **50**: 589-596.

Suenaga, T., Ota, T., Oba, K., Usui, K., Sako, T., Hori, T., *et al.* (2021) Combination of (15)N Tracer and Microbial Analyses Discloses N2O Sink Potential of the Anammox Community. *Environ Sci Technol* **55**: 9231-9242.

Throback, I.N., Enwall, K., Jarvis, A. and Hallin, S. (2004) Reassessing PCR primers targeting nirS, nirK and nosZ genes for community surveys of denitrifying bacteria with DGGE. *FEMS Microbiol Ecol* **49**: 401-417.
